# Structural Rigidity Enables Amplified Spontaneous Emission in 2D Alkylammonium Tin Perovskites

**DOI:** 10.1021/jacs.6c14883

**Published:** 2026-10-12

**Authors:** Francesca Canestra, Nurgul Sarsembek, Yarong He, Andrea Pianetti, Martina Santarelli, Silvia Contessi, Ranita Samanta, Martina Zangari, Federico Brivio, Tommaso Salzillo, Cristina Femoni, Annamaria Petrozza, Daniele Cortecchia

**Affiliations:** † Department of Industrial Chemistry “Toso Montanari”, 9296 University of Bologna, 40129 Bologna, Italy; ‡ 121451 Center for Nano Science and Technology @Polimi, Istituto Italiano di Tecnologia, via Rubattino 81, Milano 20134, Italy; § Department of Physics, Politecnico di Milano, Milano 20133, Italy; ∥ Department of Medical and Surgical Sciences DIMEC, University of Bologna, 40138 Bologna, Italy; ⊥ Department of Physics and Astronomy “Galileo Galilei”, University of Padova, Via Francesco Marzolo 8, 35131 Padova, Italy

## Abstract

Two-dimensional tin perovskites are attracting increasing attention for coherent light emission, yet the impact of organic cations on their properties remains poorly understood. Here, we compare the Ruddlesden–Popper (RP) butylammonium tin iodide (BA_2_SnI_4_) and Dion–Jacobson (DJ) 1,4-diammoniobutane tin iodide (DABSnI_4_) perovskites to investigate the impact of the two structural types on their photophysical and optical gain properties. Combining X-ray diffraction and solid-state NMR spectroscopy, we reveal the different templating properties of the mono- and ditopic cations on the long-range structural order and local coordination environment, identifying an unreported phase transition for DABSnI_4_. The constrained molecular motions in the DJ perovskite provide an increased structural rigidity that also suppresses vibrational modes of the inorganic sublattice. The consequent lower exciton–phonon coupling and exciton binding energy, reduced defectivity, and modulation of the exciton recombination dynamics create favorable conditions to stabilize the population inversion and achieve amplified spontaneous emission (ASE) in DABSnI_4_, in net contrast with BA_2_SnI_4_ where ASE could not be obtained. This work highlights that Dion–Jacobson perovskites can create more rigid structural environments with positive feedback on the photophysical properties, providing a simple but effective design strategy for the synthesis of improved materials for optoelectronic applications.

## Graphical abstract

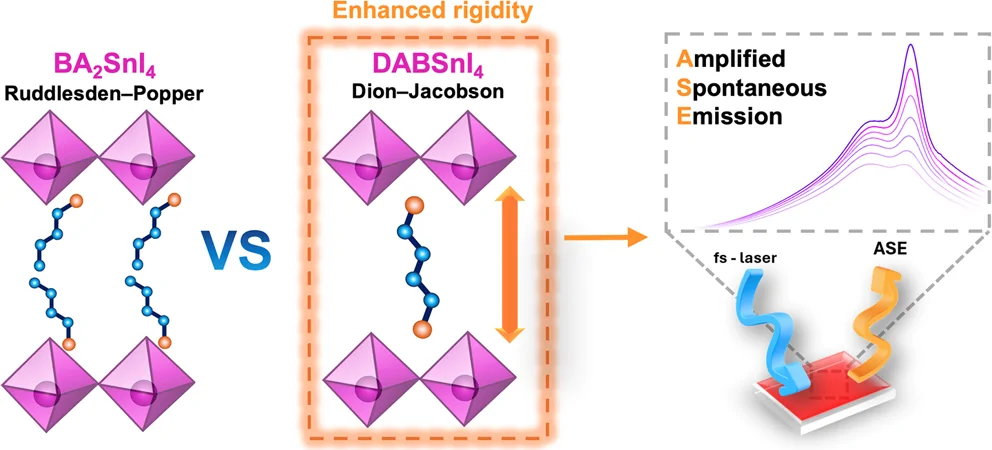

## Introduction

Metal halide perovskites are promising semiconductors for optoelectronic applications thanks to their solution processability and tunable luminescence,^1,2^ with optical and electronic properties that can be significantly modulated through the choice of constituent elements and the nature of the organic component.^3^ Since the first demonstrations of amplified spontaneous emission (ASE) and lasing in three-dimensional (3D) lead halide perovskites,^4,5^ great strides have been made fostering their photonic applications as coherent light sources,^6^ passing through the demonstration of lasing under CW optical pumping and electrically driven lasing in dual cavity devices.^7,8^ However, the need to improve the material stability against moisture uptake, oxidation and ionic diffusion and to further expand the spectral range has prompted deeper investigations of layered perovskites.^9^ Lead-based 2D perovskites had been preferentially studied since 1990s;^10–13^ however, issues in the reproducibility of their lasing performance have significantly slowed down the progress compared to 3D perovskites.^14–16^ On the other hand, tin-based 2D perovskites have recently been shown to possess better properties for coherent emission,^17^ with the first demonstration of lasing upon integration into a distributed-feedback (DFB) resonator spurring new interest in the field;^18^ since then several tin-based compositions have been shown to yield efficient ASE or lasing. These include PEA_2_SnI_4_ (PEA = phenethylammonium),^17–19^ (4FPEA)_2_SnI_4_ (4FPEA = 4-fluorophenethylammonium),^20^ NMA_2_SnI_4_ (NMA = 1-naphthylmethylammonium),^18^ (2T)_2_SnI_4_ (2T = bithiophenylethylammonium),^19^ (3T)_2_SnI_4_ (3T^+^ = 2-([2,2′:5′,2″-terthiophen]-5-yl)­ethan-1-aminium),^19^ (BrCA3)_2_SnI_4_ (2-(2-bromo-5-carboxyphenoxy)­ethan-1-aminium),^21^ (TEA)_2_SnI_4_ (TEA = 2-thiopheneethylamine),^22^ and (ThMA)_2_Cs_n‑1_Pb_n_Br_3n+1_ (ThMA = 2-thiophenemethylammonium).^23^ Notably, all these cations possess an aromatic character with π-conjugated electron systems, where π–π stacking interactions can create a rigid structural environment, which is believed to be an important factor in the radiative recombination process of the material.^17,19,24^ In some cases, like in BrCA_3_,^21^ this is further strengthened with the functionalization of the aromatic core enhancing the intermolecular interactions. On the contrary, coherent emission was not achieved with butylammonium (BA) cations in BA_2_SnI_4_ but only observed in higher dimensional systems like (BA)_2_MA_*n*‑1_Sn_*n*_I_3*n*+1_ with *n* ≥ 2.^18,19^ Similar observations were also reported, in some cases, for their lead counterpart.^25^ The worse performance of these perovskites was attributed to the defectivity and the increased flexibility of alkylammonium cations, which are missing the additional structural stabilization associated with an aromatic spacer, such as PEA, and their π–π interactions. This highlights the critical importance of the structural and compositional engineering of the material,^3^ calling for a deeper investigation of the correlation between the templating properties of the organic cation, the imparted structural rigidity, and its impact on the photophysical properties of the material, which is still only marginally understood.

Various aspects of alkylammonium perovskites have been investigated, including the thermochromic behavior,^26^ the odd–even effect on the crystal packing,^26^ the controlled preferential orientation growth in thin films,^27^ and the impact of the chain length on the dielectric confinement of the quantum well.^28^ As a representative system of mono- and ditopic alkylammonium cations, we compare BA_2_SnI_4_ (BA = butylammonium) and DABSnI_4_ (DAB = 1,4-diaminobutane) to investigate if the different binding properties of the templating cation can yield more favorable structural and photophysical properties and positively impact the material’s quality and optical gain performance. BA templates a Ruddlesden–Popper (RP) perovskite where inorganic sheets are separated by bilayers of organic cations, while DAB yields a Dion–Jacobson (DJ) architecture where the bidentate ligands directly link adjacent inorganic layers without the presence of a van der Waals gap. We identified a new low-temperature monoclinic phase of DABSnI_4_ where ASE can be achieved upon optical pumping, contrary to BA_2_SnI_4_. Combined X-ray diffraction, Raman microspectroscopy, and solid-state nuclear magnetic resonance (ssNMR) investigations provided complementary information on the long-range structural order, the local coordination environment, and molecular dynamics. The different binding capabilities of the two ligands not only markedly modify the temperature-dependent structural evolution of the perovskites but also constrain the molecular motion in the DJ perovskites, as confirmed by temperature-dependent spin–lattice relaxation measurements. This yields a more rigid structural framework and consequent suppression of the vibrational modes of the inorganic sublattice. This deeply impacts the photophysical properties, inducing a lower exciton–phonon coupling and reduced defectivity in DABSnI_4_ with respect to BA_2_SnI_4_ and the modulation of the exciton recombination dynamics. This adds to a lower exciton binding energy in DABSnI_4_ (around 50 meV), consistent with a reduction in the quantum and dielectric confinement due to the shorter interplanar distance. Overall, we find that the combination of these factors favors the realization of population inversion in DABSnI_4_, where ASE could be achieved up to 150 K, in stark contrast to BA_2_SnI_4_ where ASE conditions were not reached. These findings highlight that DJ perovskites can be more suitable alternatives than RP phases for applications in coherent light emission and provide useful guidelines for the rational design of templating cations, enhancing the structural rigidity and optoelectronic properties of layered perovskites.

## Results and Discussion

BA_2_SnI_4_ and DABSnI_4_ 2D perovskite crystals were synthesized via a slow cooling method from concentrated HI/H_3_PO_2_ solutions, and single-crystal X-ray diffraction (Figure 1a,b) was employed to investigate their structural properties. As typically observed in alkylammonium perovskites,^26^ BA_2_SnI_4_ undergoes a phase transition at low temperatures, which is well documented in the literature;^26,29–32^ two different phases were determined by Zhang et al.,^32^ at 125 and 298 K, and they both adopt an orthorhombic crystal structure (space group *Pbca*) with a marked increase in the octahedral tilt in the low-temperature phase (Figure 1a and Table S1). Here, we also further identify two different phases for DABSnI_4_: a triclinic structure (space group *P-1*) at 298 K, in agreement with a previous report by Ji et al.^33^ and Vishwajith et al.,^34^ and a newly determined monoclinic structure (space group *P*2_1_/*c*) at 100 K, with a doubling of the unit cell volume from 759 Å^3^ to 1482 Å^3^ (Figure 1b and Table S1).

**Figure 1:**
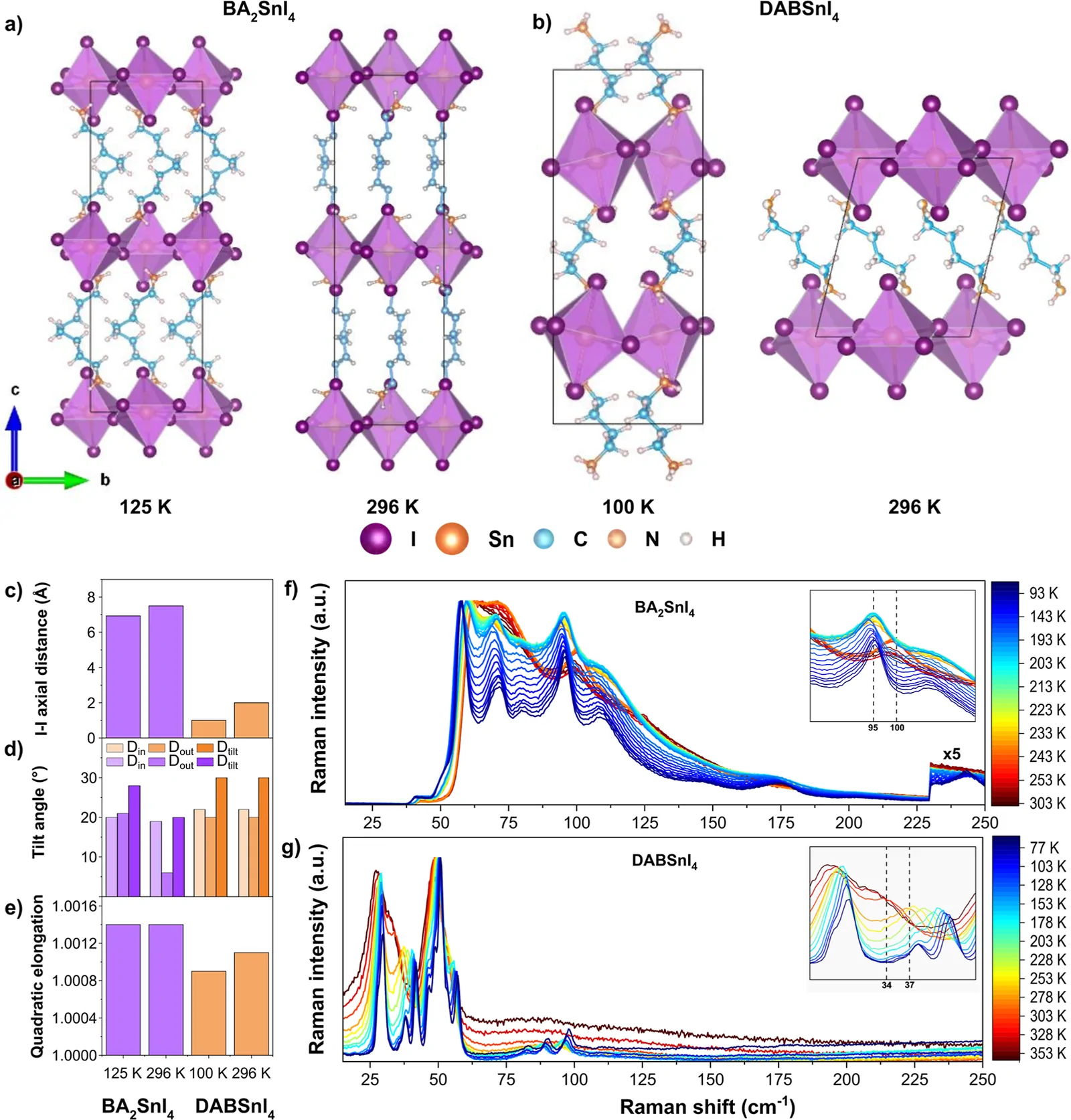
Crystal structures from single-crystal X-ray diffraction showing the low- and high-temperature phases of (a) BA_2_SnI_4_ at 125 K (structural data from ref 31) and 296 K and of (b) DABSnI_4_ at 100 and 296 K, determined in this work. Evolution of structural parameters determined from single-crystal XRD structures: (c) interplanar distances between inorganic layers (measured as the distance between iodides in the apical position), (d) in-plan tilt angle (D_in_), the out-of-plane tilt angle (D_out_), octahedral tilting (D_tilt_), and (e) quadratic elongation. See Table S3 for additional details. The structural parameters for BA_2_SnI_4_ at 125 K are calculated from the crystal structures reported in ref 32. Temperature-dependent low-energy Raman spectra of (f) BA_2_SnI_4_ measured with a 785 nm laser, with a cutoff filter at 50 cm^–1^ (after 230 cm^–1^, the wavenumbers are multiplied by a factor of 5 for clarity), and (g) DABSnI_4_, measured with 647.1 nm laser excitation. The main spectral changes occurring at the phase transitions are highlighted in the insets.

In BA_2_SnI_4_, the monovalent butylammonium (BA^+^) bilayers separate the [SnI_4_]^2–^ layers, inducing a staggered configuration between adjacent inorganic sheets characteristic of the Ruddlesden–Popper (RP) architecture (Figure S1).^35^ In contrast, the ditopic 1,4-diammoniobutane (DAB^2+^) directly links neighboring inorganic layers without the presence of a van der Waals gap, forming a Dion–Jacobson (DJ) perovskite with an eclipsed configuration and forcing a higher octahedral tilt compared to BA_2_SnI_4_ (Figure S1).^36,37^ This is reflected in shorter interplanar distances for DABSnI_4_ (3.90 Å at 100 K and 4.02 Å at room temperature (RT)) compared to those in BA_2_SnI_4_ (6.93 Å at 125 K and 7.50 Å at RT) (Table S3 and Figure 1c). The structural properties of the inorganic component were quantitatively analyzed by extracting the octahedral tilt angles (in-plane tilt D_in_, out-of-plane tilt D_out_, and tilt angle D_tilt_)^38–41^ and quadratic elongation (Table S3 and Figure 1d,e). In BA_2_SnI_4_, the phase transition is accompanied by an increase in tilt angle from high to low temperature, changing from 6° (298 K) to 21° (125 K), mostly driven by the change in D_out_. No differences are found in the quadratic elongation of 1.0014 which remains stable. On the other hand, no change in tilt angle parameters is observed for DABSnI_4_, and we observe only a very minor change in octahedral distortion, with the quadratic elongation going from 1.0011 to 1.0009 (Table S3 and Figure 1d,e). This likely reflects the more constrained environment imposed by the ditopic cation.

The structural properties were further probed via Raman microspectroscopy (Figure 1f,g). The probing of low-frequency signals using an incident laser at 647.1 nm was possible only for DABSnI_4_. For BA_2_SnI_4_, due to the presence of an absorption band at this wavelength that would cause a strong photoluminescence background in the Raman spectrum, we employed a laser at 785 nm. This also required the use of an edge filter that excluded the low-frequency region below 50 cm^–1^ (see the Experimental Section). The Raman spectra in Figure 1f,g show the vibrational modes characteristic of the inorganic part of the perovskite. Following previous assignments on MASnI_3_ ^42^ and BA_2_SnI_4_,^43^ we attribute the bands observed in the range of 45–80 cm^–1^ to Sn–I–Sn bending that causes distortion of [SnI_6_]^4–^ octahedra, while we attribute the ones at 80–120 cm^–1^ to the libration modes of the organic cations coupled to Sn–I stretching. In the case of DABSnI_4_, it was possible to extend the probed spectral range down to 20 cm^–1^ (for BA_2_SnI_4_, the spectral range could not be extended below 50 cm^–1^ due to the cutoff filter, as described in the Experimental section), highlighting the presence of a vibrational mode at 30 cm^–1^ related to the rocking and twisting motions of the inorganic octahedra.^44,45^ The vibrational frequencies are blue-shifted in BA_2_SnI_4_, suggesting the presence of a stronger metal halide interaction than in DABSnI_4_. However, the modes in the range of 80–120 cm^–1^ are strongly suppressed in DABSnI_4_, indicating that the conformational constraint of DAB^2+^ in the DJ perovskite allows to minimize the vibrational motions of the inorganic lattice; Raman modes in this range have also been previously shown to provide qualitative information about the lattice stiffness in the direction perpendicular to the perovskite plane.^46^ This is further corroborated by the fact that the high-frequency signals above 130 cm^–1^, related to the libration and torsional modes of the organic cation interacting with the inorganic cage, are silent in DABSnI_4_, while they can be clearly discerned in BA_2_SnI_4_, in line with the previous experimental studies on 2D lead perovskites.^45^ Notably, vibrations with frequencies below 300 cm^–1^ have been demonstrated to affect the nonradiative recombination in BA_2_SnI_4_;^43^ thus, we expect that their suppression may be critical to enhance the photophysical properties of the material. The temperature dependence shows a discontinuous evolution of the spectral features around 240 K in BA_2_SnI_4_ (see Figures S3 and S4), denoting the occurrence of the expected phase transition (band shift from 95 cm^–1^ to 101 cm^–1^). In DABSnI_4_, only a small discontinuous shift is observed in the signal around 37 cm^–1^ (from 278 to 303 K), consistent with the minor structural reorganization of the inorganic framework accompanying its phase transition probed via single-crystal XRD.

To comparatively elucidate how spacer chemistry governs structural organization and molecular dynamics, solid-state NMR (ssNMR) was measured on the powders of two-layered tin iodide perovskites (Figure S5). Variable-temperature ^13^C MAS ssNMR spectra were acquired across the structural phase-transition regimes characteristic of each compound. For BA_2_SnI_4_, four distinct resonances are observed for the chemically equivalent butylammonium spacer (Figure 2a). The signal at 42 ppm is assigned to the carbon nearest the ammonium headgroup, reflecting deshielding by the electronegative nitrogen. The internal methylene groups appear at 30 and 21 ppm, while the resonance at 15 ppm corresponds to the terminal methyl (CH_3_) group. The terminal CH_3_ group undergoes rapid 3-fold rotation, reflected in the slow ^1^H–^13^C cross-polarization build-up rate (Kcp = 0.55 ms^–1^, Figure S7);^47,48^ upon cooling, the progressive slowdown of this motion leads to pronounced broadening and attenuation of the CH_3_ resonance.^49^ Across this interval, the carbon adjacent to the ammonium headgroup moves to a lower chemical shift, while the methylene and methyl carbons shift to a higher chemical shift, consistent with a thermally driven structural phase transition at 243 K that modifies the local environment of the organic spacer.

**Figure 2:**
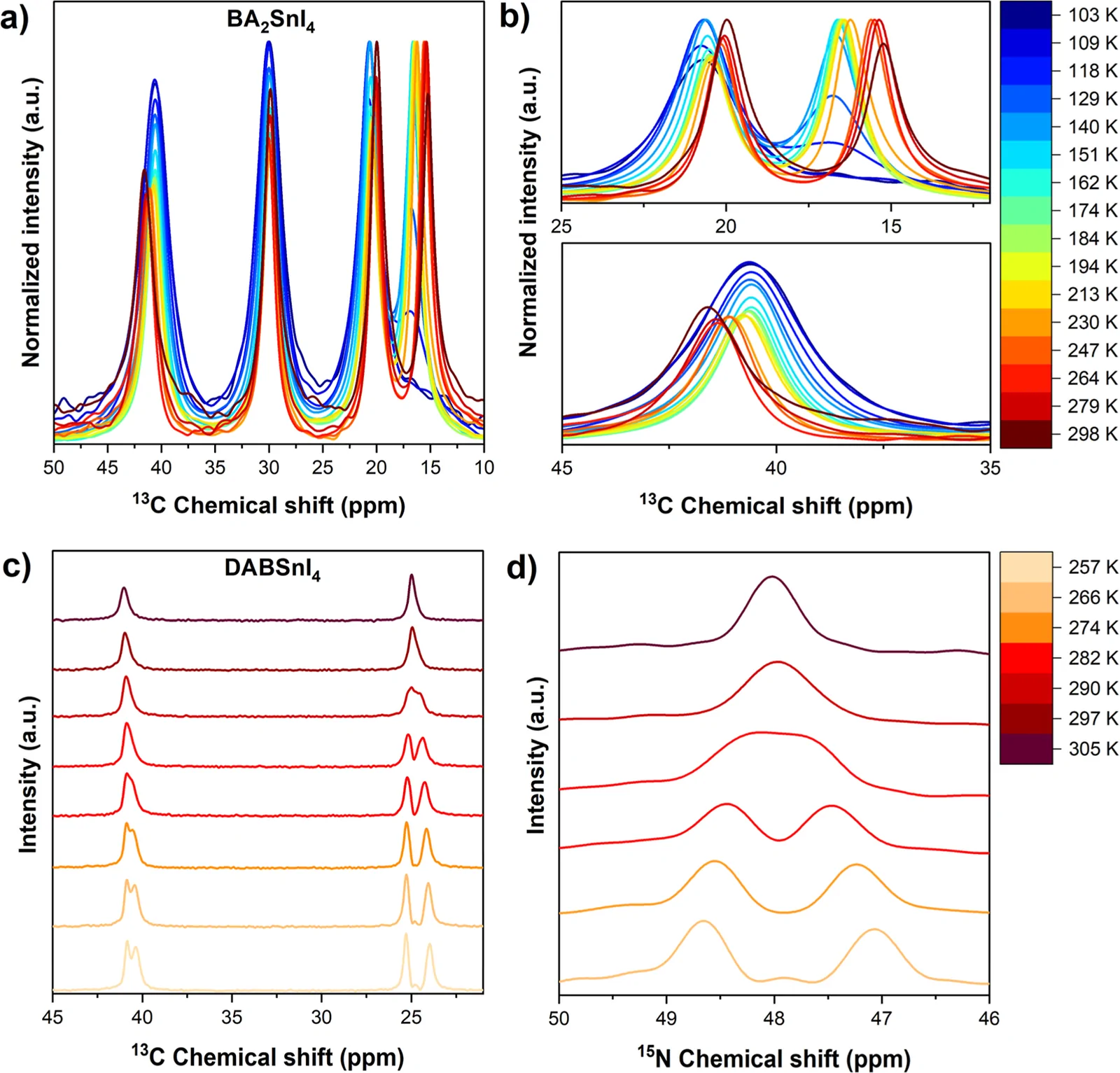
Variable-temperature ssNMR spectra of BA_2_SnI_4_ and DABSnI_4_ acquired across their respective phase transition temperatures. (a) ^13^C spectra of BA_2_SnI_4_ at the 5 kHz MAS rate and (b) expanded regions highlighting the temperature-dependent changes. (c) ^13^C MAS spectra at 15 kHz and (d) ^15^N MAS spectra at 10 kHz of DABSnI_4_.

In contrast, the directly bridged DABSnI_4_ framework displays a different spectral response. At ambient temperature, the diammonium organic spacer exhibits two ^13^C resonances: a peak at 42 ppm assigned to the two carbons adjacent to the ammonium head groups and a signal at 24 ppm corresponding to the central methylene carbons (Figure 2c), in agreement with DFT calculations (Figure S8). Upon cooling through a structural phase transition near 298 K, each of these signals split into two distinct resonances. This spectral doubling indicates a symmetry-breaking transition from the high-temperature phase and the emergence of magnetically inequivalent carbon sites within the asymmetric unit, which was also confirmed via DFT calculations of the ^13^C chemical shifts (Figure S8). This interpretation is further corroborated by variable-temperature ^15^N MAS ssNMR spectra, which reveal analogous splitting of the single ammonium resonance across the same temperature range (Figure 2d). The divergent temperature-dependent shifts of the two signals provide direct evidence for the distinct local nitrogen environments in the low-temperature DJ phase. These observations are consistent with findings previously reported in our study of the Dion–Jacobson perovskite (EDBE)­PbI_4_.^50^ The observed splitting reflects nonequivalent supramolecular arrangements of the ammonium headgroups, serving as an NMR fingerprint of the coordination environments of DJ architectures.

Building on the temperature-dependent ^13^C spectral evolution, quantitative insight into spacer rigidity is obtained from the transverse relaxation behavior. According to the relation fwhm = 1/πT_2_, the broader ^13^C line widths observed for DABSnI_4_ correspond to shorter T_2_ values arising from diminished motional averaging. Consequently, residual dipolar couplings are less efficiently averaged by molecular motion, leading to accelerated phase dephasing and enhanced spin–spin relaxation.^51^ The markedly shorter T_2_ values observed for DABSnI_4_ across the investigated temperature range therefore indicate a significantly more rigid organic framework compared to BA_2_SnI_4_ (Figure 3a, b). In BA_2_SnI_4_, the butylammonium spacer exhibits a gradient of motional freedom along the alkyl chain, with greater mobility at the terminal methyl group, as shown by its low Kcp value and progressively increased rigidity toward the ammonium headgroup anchored to the inorganic layer through N–H···I hydrogen bonding (Figure 3a). In contrast, the bivalent diammonium spacer is anchored at both ends to the inorganic framework, strongly coupling the organic and inorganic sublattices and restricting internal degrees of freedom. As the structural phase transition temperature is approached, T_2_ progressively decreases and reaches a minimum, indicating the regime of maximum restriction of molecular motion. After the symmetry-breaking transition, T_2_ increases slightly but remains significantly shorter than in BA_2_SnI_4_, reflecting the persistently more constrained dynamics of the diammonium framework (Figure 3b).

**Figure 3:**
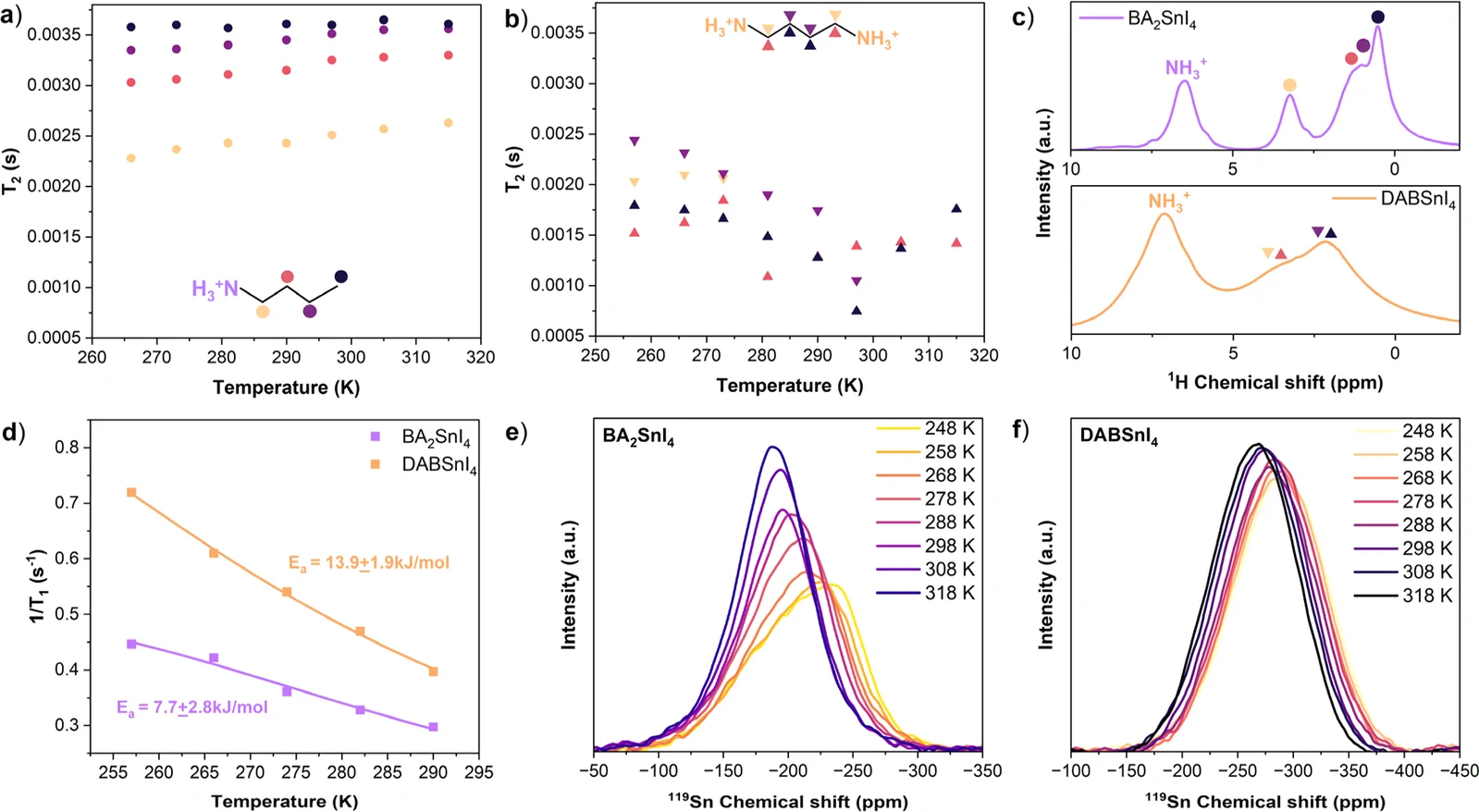
Comparison of the structural rigidity of BA_2_SnI_4_ and DABSnI_4_ probed by ssNMR. Temperature-dependent ^13^C transverse relaxation times (T_2_) of (a) BA_2_SnI_4_ and (b) DABSnI_4_ with markers indicating chemically distinct carbon environments. (c) ^1^H spectra at the 42 kHz MAS rate of BA_2_SnI_4_ (top) and DABSnI_4_ (bottom). (d) Temperature-dependent ^1^H spin–lattice relaxation times (1/*T*_1_) for BA_2_SnI_4_ (orange) and DABSnI_4_ (purple). Variable-temperature ^119^Sn static ssNMR spectra of (e) BA_2_SnI_4_ and (f) DABSnI_4_.

The ^1^H MAS ssNMR spectra at 42 kHz provide complementary information on the proton environments of the organic spacers (Figure 3c). Owing to the dense ^1^H–^1^H dipolar network and efficient spin diffusion, proton magnetization rapidly equilibrates across the molecular framework. Consequently, ^1^H relaxation reflects an averaged response of the proton ensemble rather than site-specific dynamics, and the spectra for DABSnI_4_ remain largely unchanged across the investigated temperature range as the substantial line broadening masks subtle spectral changes associated with the phase transition. In both systems, the most downfield ^1^H resonance is assigned to the ammonium protons at 6.65 ppm. The adjacent methylene protons appear at the next more deshielded downfield position at 3.2 ppm in BA_2_SnI_4_,^47^ while in DABSnI_4_, they contribute to a broader feature centered around 3.6 ppm. Similarly, the remaining alkyl protons appear at lower chemical shifts, with BA_2_SnI_4_ showing a signal near 0.5 ppm arising from overlapping internal CH_2_ and terminal CH_3_ groups,^47^ while in DABSnI_4_, the central ethylene protons are observed around 2 ppm. To further investigate these dynamics, the corresponding ^1^H spin–lattice relaxation times (*T*_1_) were analyzed using the Bloembergen–Purcell–Pound (BPP) model^52^ (Figure 3d; also see SI for further details). Analysis within the temperature window relevant to the optical characterization (256–290 K) yields an activation energy (Ea) of 13.9 ± 1.9 kJ/mol for DABSnI_4_, significantly higher than the value extracted for BA_2_SnI_4_ (7.7 ± 2.8 kJ/mol). This increased barrier confirms more strongly hindered proton dynamics in the bivalent system DABSnI_4_, confirming that bifunctional anchoring effectively suppresses molecular reorientation and conformational disorder.

The influence of organic dynamics on the inorganic framework is directly reflected in the ^119^Sn static ssNMR response. In BA_2_SnI_4_, the chemical shift anisotropy (CSA) increases markedly upon cooling from 32 to 66 ppm, accompanied by a significant isotropic chemical shift change and an asymmetry parameter from 0.953 to 0.498 (Figure 3e and Table 1). This evolution indicates an increased distortion within the SnI_6_ framework and reflects more conformational disorder of the organic cation in the RP phase due to the larger motional freedom. Conversely, DABSnI_4_ exhibits only subtle temperature-dependent changes in the ^119^Sn tensor parameters and a minor chemical shift variation near the phase-transition temperature. Meanwhile, the CSA remains nearly invariant (51–55 ppm) despite the occurrence of the phase transition, consistent with a structurally constrained local environment around tin nuclei (Figure 3f).

**Table 1:** ^119^Sn Isotropic Chemical Shifts (δ_iso_), CSA (Δδ), and Asymmetry Parameter (η) Defined According to the Haeberlen Convention

sample	T (K)	^119^Sn, δ_iso_ (ppm)	Δδ (ppm)	η
BA_2_SnI_4_	318 K	–188	32.58	0.953
	248 K	–213	65.70	0.498
DABSnI_4_	318 K	–246	54.46	0.906
	248 K	–275	50.48	0.937

The photophysical properties were probed on thin films of the two perovskites (see Figure S6). UV–vis absorption spectra of both films show the sharp excitonic absorption arising from the quantum and dielectric confinement characteristic of 2D perovskites, followed at higher energies by the absorption continuum. For BA_2_SnI_4_, the excitonic peak appears at 603 nm, while for DABSnI_4_, it is blue-shifted to 571 nm. Upon photoexcitation, both materials emit red narrowband photoluminescence, with emission peaks at 622 nm for BA_2_SnI_4_ and 607 nm for DABSnI_4_ (Figure 4a). The observed blue shift in both absorption and emission for DABSnI_4_ is attributed to its greater octahedral tilting (Table S3). This increased structural distortion reduces the orbital overlap between tin and iodide ions, leading to a widening of the bandgap. The more pronounced tilting in DABSnI_4_ reduces the overlap between the iodine 5p and tin 5s orbitals, effectively increasing the bandgap and resulting in the observed blue shift in both absorption and emission spectra.^53,54^ Photoluminescence (PL) measurements as a function of temperature were carried out for both perovskites (Figure 4b,c), from 77 to 333 K. Here, a noticeable red shift of 41 nm in the PL peak position was observed for BA_2_SnI_4_, at approximately 243 K (see Figure S13), while no evident shift appears in DABSnI_4_. This is also backed by the temperature dependence of their absorption spectra (Figure S9), and it is consistent with the evolution of the octahedral tilts upon phase transition, significantly changing in BA_2_SnI_4_ while remaining constant in DABSnI_4_ (Figure 1d and Table S3).

**Figure 4:**
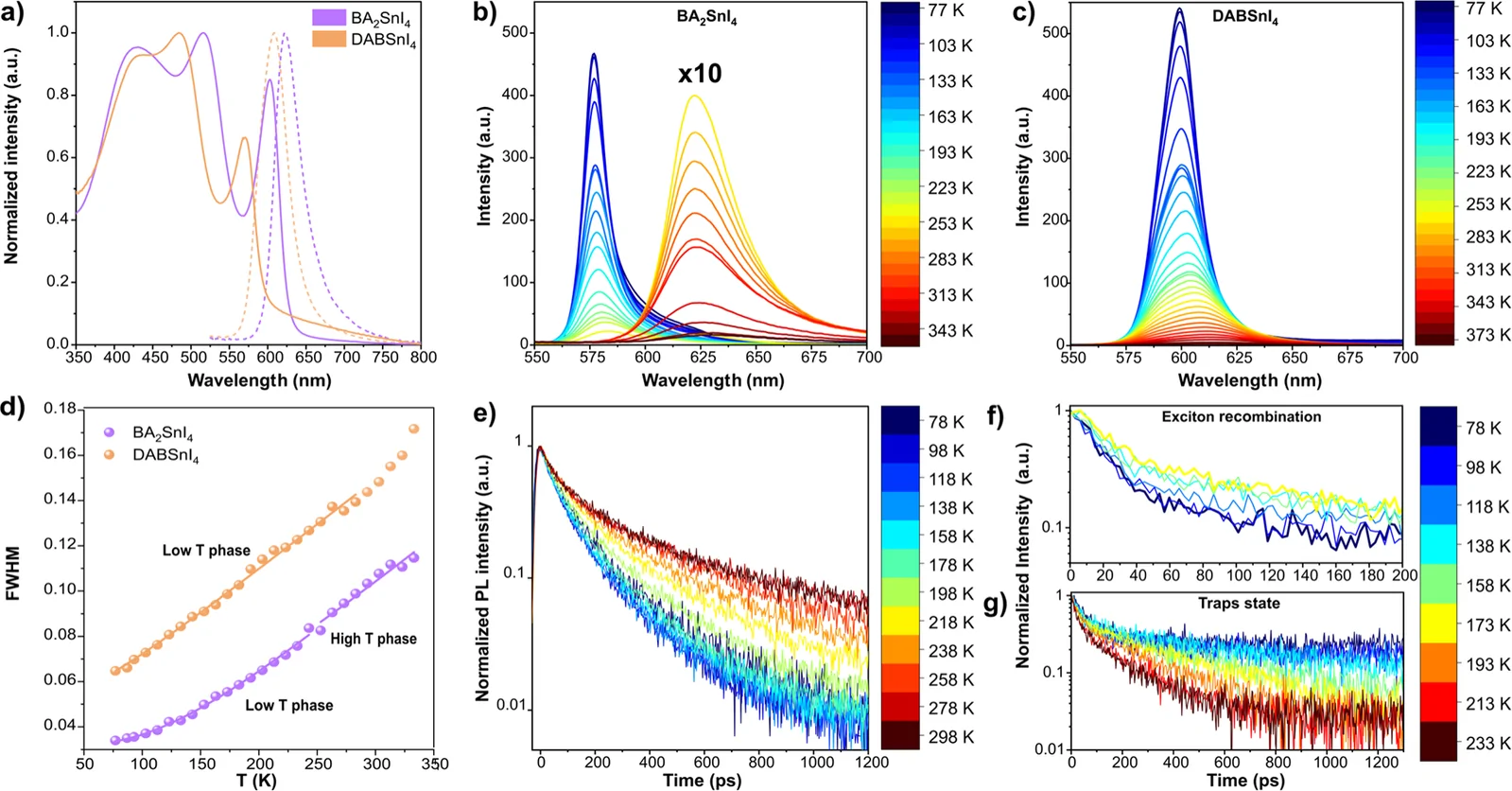
Photophysical characterization of BA_2_SnI_4_ and DABSnI_4_. (a) Absorption and photoluminescence (PL, dotted lines) spectra of both perovskites, showing a blue shift for DABSnI_4_. (b) Temperature-dependent PL spectra (exciting with a blue 405 nm laser in both cases) of BA_2_SnI_4_ from 77 to 298 K, with a clear phase transition at 240 K; the PL intensity after the phase transition was multiplied by 10 to highlight the trap-related emission tail at a low temperature. (c) Temperature-dependent PL spectra of DABSnI_4_ over the same temperature range, where no evident phase transition is observed. (d) Temperature dependence of the PL full width at half-maximum (fwhm) of the excitonic peak centered around 577 nm for BA_2_SnI_4_ and 599 nm (2.15 and 2.07 eV, see Figures S10 and S11) with corresponding fitting curves: the fit for BA_2_SnI_4_ is divided before and after the phase transition temperature (243 K), while DABSnI_4_ is fitted only for the low-temperature phase before 283 K. Time-resolved photoluminescence (TRPL) for (e) DABSnI_4_, (f) the excitonic recombination of BA_2_SnI_4_ (integration range from 500 to 558 nm), and (g) the trap emission of BA_2_SnI_4_ (integration range from 600 to 700 nm).

In the case of BA_2_SnI_4_, the excitonic emission is followed by an evident low energy tail arising from trap states centered around 601 nm (2.06 eV), which in DABSnI_4_ is significantly less prominent compared to the excitonic emission (see the details on signal deconvolution in Figures S10–S15).

For the quantitative analysis of the mechanism underlying photoluminescence broadening in the two materials, we evaluated the temperature-dependent evolution of the full width at half-maximum (fwhm) of the main excitonic emission at 577 nm (2.15 eV) in BA_2_SnI_4_ and 599 nm (2.07 eV) in DABSnI_4_ (Figure 4d). In both perovskites, the fitting considers the presence of a phase transition. Assuming line width broadening to be mostly affected by the Fröhlich interaction of excitons with longitudinal optical (LO) phonons, the exciton–phonon coupling strength can be described by the following expression:^19,55,56^

1 $$\Gamma \left(T\right)=\Gamma_{inh}+\frac{\Gamma_{LO}}{e^{\left(E_{LO}/k_{B}T\right)}-1}$$

where Γ_inh_ is the inhomogeneous peak width at zero temperature, Γ_LO_ is the exciton-longitudinal optical (LO) phonon coupling coefficient, k_B_ is the Boltzmann constant, and E_LO_ is the LO phonon energy. The fittings (see Table S7) were obtained with the phonon energies E_LO_ = 12 meV for DABSnI_4_ and E_LO_ = 32 meV for BA_2_SnI_4_, corresponding to the Raman modes at 97 cm^–1^ and 245 cm^–1^, respectively (Figures 1f,g and S2). The higher-energy mode associated with the LO phonon in BA_2_SnI_4_ suggests that the inorganic framework is strongly influenced by the organic moieties. In contrast, the ditopic cation in DABSnI_4_ appears to provide an increased structural rigidity also to the inorganic sublattice. This highlights that the libration modes of the DAB^2+^ cations coupled to Sn–I stretching and the torsional motion of BA^+^ directly affect the radiative recombination of the materials.^43^ This is further corroborated by the weaker exciton phonon coupling in DABSnI_4_ (Γ_LO_ = 57 ± 9 meV) compared to BA_2_SnI_4_ (Γ_LO_ = 175 ± 29 meV below and 163 ± 14 meV above 243 K); a larger value of Γ_LO_ broadens more significantly the emission bandwidth with increasing temperature and enhances nonradiative decay channels.^57^ Notably, we find that the lower Γ_LO_ of DABSnI_4_ corresponds to a higher activation energy of the molecular motion determined through the modeling of the ssNMR data. This further confirms how the constrained cation led to a more rigid environment that in return minimized the exciton–phonon coupling in DJ perovskites. From the analysis of photoluminescence thermal quenching, we further retrieved the exciton binding energy (*E*_b_) of the perovskites, as shown in Figure S16.^17,58–60^ The calculated Eb values are 65 ± 6 meV for BA_2_SnI_4_ and 46 ± 3 meV for DABSnI_4_ (Table S7). The values align well with the weaker intensity of the excitonic absorption in DABSnI_4_ and with the shorter interlayer distance of the DJ perovskite (see Figure 1c and Table S3), which is expected to alleviate the quantum and dielectric confinement in the multi-quantum-well structure of the 2D perovskite, thus reducing the E_b_.^28,61^ Exciton recombination dynamics were investigated via time-resolved photoluminescence (TRPL, Figures 4e–g and S17–S20). The PL of DABSnI_4_ shows an almost perfect monoexponential decay at 78 K: increasing the temperature leads to a progressively more stretched shape of such decay and consequently a higher mean lifetime of emission (Figure 4e); this peculiar trend is the also observed in PEA_2_SnI_4_ (PEA = phenethylammonium),^17,60^ where it was previously attributed to dark excitonic states located close to the lowest radiative exciton and acting as a reservoir for the excited-state population at higher temperatures.^60^ To a smaller extent, the excitonic emission of BA_2_SnI_4_ also follows a similar increase of lifetime with temperature (Figure 4f); however, its behavior is complicated by the convolution with the lower energy and long-lived emission from trap states, having an opposite decreasing trend of the lifetime with temperature (Figure 4g). To highlight this behavior, the evolution of the excitonic TRPL decay of BA_2_SnI_4_ in the range of 500–558 nm is plotted in Figure 4f only up to 173 K (at which the excitonic emission can still be sufficiently spectrally resolved), while the TRPL evolution of the trap states in the spectral range of 600–700 nm is shown in Figure 4g for the low-temperature phase of BA_2_SnI_4_ (78–233 K); the entire temperature range up to 293 K is shown in Figure S20. The excitonic emission quickly decays within 200 ps (Figure 4f), while the low energy component is much longer lived, extending beyond 1 ns, (Figure 4g) and shows a decrease of lifetime with temperature, in contrast with the excitonic part having the opposite trend. This supports the different nature of the low energy emissive states and points to the presence of trap states that partially quench the excitonic emission. Moreover, the relative contribution from trap emission increases with temperature (see also Figure S12), suggesting the presence of a thermally activated trapping mechanism that increasingly quenches the excitonic luminescence particularly at temperatures beyond 200 K. This results in a strong overlap between excitonic and trap-related contributions as temperature increases, with both the temporal decay and the temperature dependence of the PL intensity, width, and shift reflecting their combined effects (Figures S10, S11, and S20). Overall, the analysis indicates a stronger involvement of trap states in the photoluminescence recombination dynamics of BA_2_SnI_4_ compared to DABSnI_4_; this also aligns well with our previous characterization of trap states in BA_2_SnI_4_ via photothermal deflection spectroscopy.^18^

Finally, we find that the different structural and photophysical properties affect the ability of the two materials to sustain ASE. ASE is a cooperative optical-gain phenomenon that precedes full lasing; it arises when spontaneously emitted photons are repeatedly amplified while traversing an optical medium. Because ASE does not require a resonant cavity, the observed threshold fluence is a representative parameter to investigate the gain of the material in relation to its intrinsic properties and their effect on the balance between stimulated emission and competing nonradiative decay channels.^18,62^ Notably, from the fluence-dependent photoluminescence spectra recorded at 78 K, we were unable to reach the conditions for ASE in BA_2_SnI_4_ (Figure 5a) also probing different film morphologies (Figure S22), in agreement with our previous observations.^18^ On the other hand, a superlinear increase of a narrower ASE signal at 615 nm was clearly observed on top of the spontaneous emission for DABSnI_4_, above the threshold of 24.8 μJ/cm^2^ (Figure 5b,c). We found that the DJ perovskite forms large grains in the range of 10–50 μm which tend to pack more closely compared to BA_2_SnI_4_ (Figure S23), which can help to minimize optical losses favoring the gain buildup. Hyperspectral photoluminescence maps of DABSnI_4_ above the excitation threshold showed the ASE to leak preferentially at the grain boundaries, likely arising from a waveguiding effect (Figure 5d,e). Despite the exponential increase of the ASE threshold with temperature, the material was able to sustain the ASE up to 150 K (Figures 5f and S21) and, to the best of our knowledge, it represents the first demonstration of ASE in a 2D alkylammonium tin perovskite. Unlike previously reported tin-based perovskite, such as PEA_2_SnI_4_,^19^ which benefit from π–π stacking between phenyl rings, alkylammonium cations lack such interactions, showing that the ditopic cation in DJ perovskites provides sufficient structural rigidity to sustain ASE. Overall, from our study, it is clear how the presence of ASE in DABSnI_4_ and not in BA_2_SnI_4_ is connected to a set of properties that are ultimately due to the templating properties of the organic cation. The ditopic nature of DAB^2+^ imparts an increased structural rigidity that suppresses relevant lattice vibrations and leads to a notable 3-fold decrease in the exciton–phonon coupling strength with respect to BA_2_SnI_4_; we identify this as the most direct photophysical consequence of the greater structural rigidity of DABSnI_4_ and as the dominant contribution to its improved optical-gain properties. This finding is consistent with other studies showing that lattice and spacer rigidity in two-dimensional perovskites controls low-frequency octahedral motions, exciton-polaron formation, and carrier relaxation^63^ and that suppression of electron–phonon coupling can enhance radiative recombination and ASE.^19,24,64^ However, other factors also come into play to shape the overall photophysical properties. Previous works have highlighted that structural rigidity can influence the photophysical activity of defects by limiting the lattice reorganization associated with carrier localization.^65^ In line with this, we observed that DAB^2+^ lowers the contribution of trap states compared to BA^+^ cations. However, due to the thermally activated nature of the trapping mechanism in BA_2_SnI_4_, we expect them to have only a marginal influence over the 78–150 K range in which ASE was probed. The shorter interplanar distance of DAB^2+^ also relieves quantum and dielectric confinement effects, leading to a lower exciton binding energy in DABSnI_4_ (46 ± 3 meV) compared to BA_2_SnI_4_ (65 ± 6 meV), a modest change compared to the exciton–phonon coupling strength. Both binding energies remain substantially larger than the thermal energy throughout the temperature range of the ASE measurements, indicating that excitons remain thermally stable in both materials and that this difference is unlikely to be the primary origin of their markedly different optical-gain properties, although it may assist the reduction in Auger recombination rate and stabilization of the excited-state population under high excitation densities.^17^

**Figure 5:**
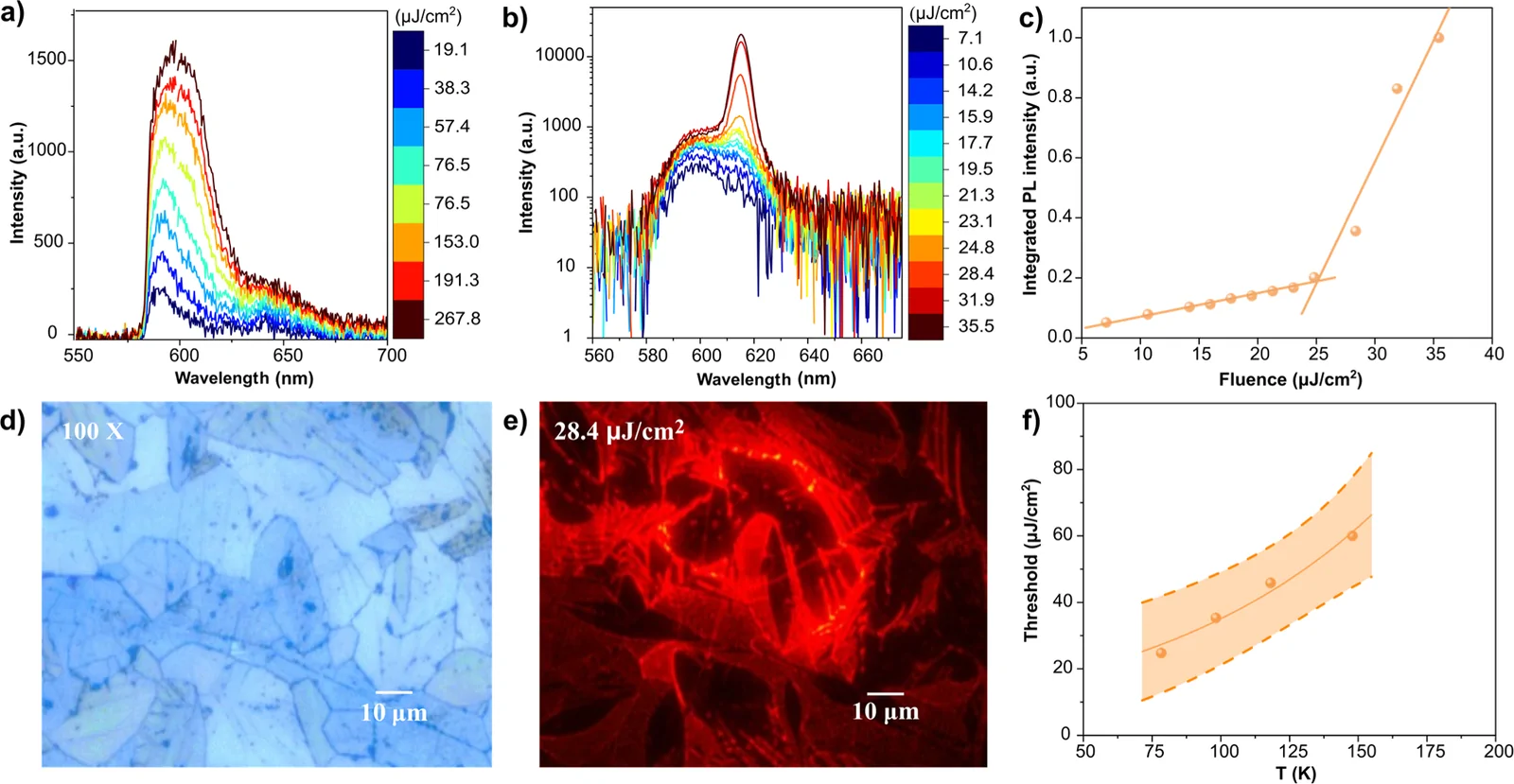
ASE evolution in BA_2_SnI_4_ and DABSnI_4_ thin films at 78 K (optical pump condition: λ_exc_ = 515 nm, repetition rate of 2 kHz, and pulse duration of 300 fs). (a) ASE of a 0.6 M thin film of BA_2_SnI_4_ at 78 K. (b) ASE of a 1 M thin film of DABSnI_4_ at 78 K, with the same pump conditions. At the low temperature, (c) the ASE threshold of DABSnI_4_ is 24.8 μJ/cm^2^. Optical images (×100 zoom) with (d) white light and (e) green light of the DABSnI_4_ thin film. (f) DABSnI_4_ can achieve the ASE until 150 K.

## Conclusion

In this work, we compared the Ruddlesden–Popper (RP) perovskite BA_2_SnI_4_ and the Dion–Jacobson (DJ) perovskite DABSnI_4_, employing the monovalent butylammonium (BA) and divalent 1,4-diammonioutane (DAB). Combining X-ray diffraction, solid-state NMR, and optical spectroscopy, we probed the different temperature-dependent structural and photophysical evolution of the two materials, identifying a new phase transition occurring near RT for DABSnI_4_, leading to a previously unreported monoclinic phase, mostly impacting the arrangement of the organic cations with minimal effects on the inorganic layers: despite the structural rearrangement of the organic sublattice, the octahedral tilt remains 30° after phase transition. This stabilization of the inorganic framework derives from the ability of the ditopic cation to lock the distortion and helps to preserve the optical properties across the phase transition. Temperature-dependent ssNMR highlighted that molecular motions are significantly more constrained in the DJ perovskites: BPP analysis of the ^1^H spin–lattice relaxation times allowed us to quantify the activation energy for molecular motions at 13.9 ± 1.9 kJ/mol in DABSnI_4_ and 7.7 ± 2.8 kJ/mol in BA_2_SnI_4_. As confirmed via Raman spectroscopy, this has repercussions also in the vibrational modes of the inorganic sublattice, providing a more rigid structural framework in DJ with respect to the RP structure. The suppression of the libration and torsional modes of the organic cations coupled to Sn–I stretching allows us to minimize the exciton–phonon coupling strength in DABSnI_4_ (57 ± 9 meV, compared to BA_2_SnI_4_ with Γ_LO_ of 175 ± 29 below 243 K and 163 ± 14 meV above 243 K), with benefits for its radiative recombination. Interestingly, DAB yields a minimal interplanar distance (3.9 Å) between adjacent inorganic layers that reduces the dielectric screening and exciton binding energy down to 46 meV, which is expected to lower the rate of Auger recombination and favor the radiative recombination at high excitation densities.^17,66–71^ The cation flexibility further affects the defectivity and exciton recombination dynamics of the materials, with traps having a deeper impact on the recombination dynamics of BA_2_SnI_4_. Through power-dependent photoluminescence measurements, we were able to probe ASE in DABSnI_4_ up to 150 K, in stark contrast with BA_2_SnI_4_ where we could not reach ASE conditions even at 78 K. We identify the 3-fold reduction in exciton phonon coupling strength as the dominant contribution to its improved optical-gain properties, further supported by the reduction of exciton binding energy and defectivity in DABSnI_4_; the increase of PL lifetime with temperature, also previously observed in PEA_2_SnI_4_,^17,60^ additionally helps to stabilize the excited-state population at higher temperatures, allowing to observe ASE up to 150 K. To the best of our knowledge, this is the first time that ASE is observed in a 2D perovskite based on a nonaromatic aliphatic ammonium cation, showing that an aromatic spacer is not intrinsically required to achieve optical gain, provided that sufficient structural rigidity can instead be established through alternative strategies. Our work indicates that DJ architectures can provide two complementary advantages that may be targeted through structural engineering to stabilize the optoelectronic properties of 2D perovskites: increased structural rigidity and the ability of the organic dication to lock the octahedral tilt. Such a strategy not only is relevant for coherent light emission but could also be transferrable to other optoelectronic and photovoltaic applications.

## Experimental Section

### Perovskite Synthesis

The synthesis of butylammonium tin iodide perovskite (BA_2_SnI_4_) was conducted under an inert atmosphere to ensure the exclusion of oxygen and moisture. In a nitrogen-filled glovebox, 223 mg of tin­(II) oxide (SnO, Great cell Solar Materials, CAS 21651-19-4) was accurately weighed and transferred to a 10 mL round-bottom flask (RBF), which was then securely closed to maintain an inert environment. The SnO powder was dissolved in 2.5 mL of a hydroiodic acid (HI, 57% w/w, Sigma-Aldrich, CAS 10034-85-2) and hypophosphorous acid (H_3_PO_2_, Sigma-Aldrich, CAS 6303-21-5) solution in a 1:1 volumetric ratio. Separately, 392 μL of butylamine (BA, C_4_H_12_N^+^, Sigma-Aldrich, CAS 109-73-9) was combined with 1 mL of the same HI/H_3_PO_2_ solution. The BA solution was added dropwise to the SnO-containing solution under continuous stirring and heating at 100 °C. After the complete addition of the BA solution, the reaction mixture was maintained at 100 °C for 5 min to ensure homogenization and reaction progress. The RBF was covered with an aluminum foil to ensure homogeneity of the temperature along the walls of the flask. Controlled crystallization was achieved by cooling the reaction mixture at a linear rate of 15 °C per hour, starting from 100 °C and continuing until RT was reached. The reaction was carried out in accordance with the stoichiometric ratio of 2:1 (2 mol of BA to 1 mol of SnO). The purification of BA_2_SnI_4_ crystals required careful handling to minimize oxidation. Following crystallization, the black crystals were rapidly filtered and washed while protecting the crystals from oxidation blowing nitrogen from the top of the Buchner filter. The optimal washing procedure involved rinsing the crystals first with a few drops of hexane, followed by diethyl ether (Et_2_O), with both solvents precooled in a freezer at −5 °C to minimize the solubility of the perovskite. After washing, the crystals were dried under vacuum and subsequently annealed at 30 °C for 60 min to ensure complete removal of residual solvents and stabilization of the material. The synthesis of diaminobutane (DAB, C_4_H_14_N_2_^+^, Thermo Scientific, CAS 110-60-1) crystals and DABSnI_4_ crystals was conducted following a procedure analogous to that used for BA, with the reaction stoichiometry in this case being 1:1.

### Thin-Film Preparation

The preparation of thin films via spin coating was analogous for both BA_2_SnI_4_ and DABSnI_4._ Square glass substrates were cleaned sequentially with distilled water, acetone, and isopropanol (IPA), using a sonicator for 5 min for each solvent. After each washing step, the substrates were dried with compressed air to prevent the formation of halos on the glass surface, which could compromise the quality of the film. The edges of the glass substrates were masked with thermoresistant tape to facilitate encapsulation and were subsequently placed in a plasma cleaner to remove any residual contaminants not eliminated during the washing process. A solution of 0.6 M was prepared inside the glovebox combining SnI_2_ (TCI, CAS 10294-70-9) in 500 μL of DMF (Sigma-Aldrich, CAS 68-12-2), with BAI for BA_2_SnI_4_. A 1 M solution of DABSnI_4_ was prepared in 500 μL of DMF, using 0.3 mol of DABI_2_ and SnI_2_. A volume of 60 μL was accurately extracted using a micropipette and deposited uniformly onto the glass substrates. The spin-coating process was conducted at 5000 rpm for 30 s to ensure homogeneous film formation, followed by thermal annealing at 100 °C (for BA_2_SnI_4_) and 150 °C (for DABSnI_4_) for 10 min to enhance crystallization. After annealing, the thermal tape was carefully removed, and the film was encapsulated between two glass substrates using a UV-curing resin (PERMABOND UV 625) to preserve an inert atmosphere and protect the film from environmental degradation.

### Single-Crystal Diffraction Analysis

Crystal data and collection details for DABSnI_4_ are reported in Table S2 in Supporting Information The diffraction experiments were carried out on a Bruker APEX II diffractometer equipped with a PHOTON2 detector using Mo–Kα radiation. Data were corrected for Lorentz polarization and absorption effects (empirical absorption correction SADABS).^72^ Structures were solved by direct methods and refined by full-matrix least-squares based on all data using F2.^72^ Hydrogen atoms were fixed at calculated positions and refined by a riding model. All non-hydrogen atoms were refined with anisotropic displacement parameters. In the case of the triclinic phase, the DAB was slightly disordered and therefore split into two positions, to achieve a better crystallographic model. Cambridge Crystallographic Data Centre (CCDC) 2559255 and 2559528 contain the supplementary crystallographic data for DABSnI4 in its monoclinic and triclinic phases, respectively. These data can be obtained free of charge from the CCDC www.ccdc.cam.ac.uk/data_request/cif.

### Raman Microspectroscopy Measurements

The Raman spectra of BA_2_SnI_4_ were acquired with a confocal Raman microscope (Horiba Xplora Plus, Horiba) equipped with edge filters enabling measurements as close as 50 cm^–1^ to the Rayleigh line. A 785 nm diode laser was used as the excitation source, with reduced power at the sample to minimize thermal damage. Each spectrum, focused on the 50–250 cm^–1^ range, resulted from a 20-s accumulation, chosen to accurately determine Raman peak positions and improve the signal-to-noise ratio. Instead, the Raman spectra of DABSnI_4_ were collected with a Jobin Yvon T6400 triple-monochromator spectrometer interfaced with an Olympus BX40 microscope in confocal configuration. The Raman excitation was from a krypton laser tuned at 647.1 nm, keeping the power at the sample of the order of 1–2 mW to avoid sample damage. Each spectrum, focused on the 5–250 cm^–1^ range, resulted from a 10-s accumulation, chosen to accurately determine Raman peak positions and improve the signal-to-noise ratio. All spectra were collected through a 20× LWD (long-working-distance) objective with a grating of 1800 grooves/mm. Raman measurements were performed on perovskite crystals. The samples were measured inside a Linkam (HFS350EV-PB4) heating/cooling stage.

### Solid-State Nuclear Magnetic Resonance (ssNMR)

Solid-state NMR measurements were performed on a Bruker Avance NEO WB with the wide-bore magnet (89 mm) operating at a 1H frequency of 400 MHz. The chemical shift was calibrated with reference to the ^13^C signal of adamantane at 37.77 ppm. A 1.9 mm cross-polarization-magic angle spinning (CP-MAS) probe with spinning frequency up to 42 kHz was used for the characterization, employing 1.9 mm zirconia rotors with VESPEL turbines. A one-dimensional ^13^C­{^1^H} and ^15^N­{^1^H} spectrum was obtained using cross-polarization, where magnetization was first excited in the ^1^H nuclei and then transferred to the targeted heteronuclear. During data acquisition, heteronuclear decoupling was applied using the SPINAL64 sequence. For ^13^C measurements, the spin rotation frequency was set to 15 kHz, for ^1^H to 42 kHz for all samples, and for ^15^N to 10 kHz for DABSnI_4_. The ^13^C CP contact time was set to 2 ms for all samples, the ^13^C 90° pulse length was 6.2 μs, and the ^1^H 90° pulse lengths were 3.32 μs for BA_2_SnI_4_ and 1.66 μs for DABSnI_4_. For DABSnI_4_, the ^15^N 90° pulse length was 9 μs, with a CP contact time of 3.5 ms. The ^1^H spin–lattice relaxation *T*_1_ at temperatures in the range of 260–330 K was measured with an inversion recovery pulse sequence under MAS at 15 kHz. Temperature calibration of the sample under magic angle spinning conditions was performed by measuring the spin–lattice relaxation time (*T*_1_) of ^79^Br in KBr via an inversion recovery experiment. ^119^Sn static spectra were acquired with a Hahn-echo pulse sequence with a 90° pulse duration of 2.3 μs, an echo delay of 12.5 μs, and accumulation of 11,008 transients for BA_2_SnI_4_ and 30,720 scans for DABSnI_4_. Line shape analysis and determination of isotropic chemical shift (δ_iso_), CSA (Δδ), and asymmetry index (η) were performed with the SOLA software package implemented in TopSpin. A 3.2 mm low-temperature magic angle spinning (LT-MAS) probe with spinning frequency up to 24 kHz was used for the characterization of BA_2_SnI_4_ for low temperature measurements, employing 3.2 mm zirconia rotors and turbines. The probe temperature was controlled by an LT-MAS cooling cabinet; the system enables sample cooling down to 80 K. The spin rotation frequency was set to 5 kHz. The ^13^C CP contact time was 3.9 ms, with ^13^C and ^1^H 90° pulse lengths of 6.2 and 4.54 μs, respectively.

### Temperature-Dependent UV–Vis Spectroscopy

Steady-state absorption and emission spectra were measured on 2D perovskite thin films deposited on glass in a Linkam HFS350EV-PB4 heating/cooling stage (under vacuum with liquid nitrogen) using an ARKEO-PL system (Cicci Research s.r.l.) composed of a CCD-based spectrometer 300–1100 nm. Optical excitation sources are composed of TTL-modulated LED and lasers with custom wavelength and direct emission with a 40° angle to the sample surface. Acquisition is performed with 600 μm fiber in reflection mode and perpendicular to the sample surface.

### Time-Resolved Photoluminescence

TRPL measurements were recorded using a Hamamatsu streak camera with a resolution of 4 to 8 ps going from TR1 to TR4. The samples were excited at 520 nm by using the frequency-doubled output of a Chameleon (Coherent) ultrafast oscillator with a pulse duration of 30 fs and an 85 MHz repetition rate.

### Amplified Spontaneous Emission Measurements

The samples were mounted on a Linkam stage under a nitrogen atmosphere. For fluence-dependent photoluminescence (PL) measurements at low temperatures, excitation was provided by a femtosecond laser (1030 nm, pulse duration ≈300 fs, Pharos, Light Conversion), which was frequency-doubled to 515 nm using an external harmonic generator (Hiro, Light Conversion). The resulting beam was collimated via a fiber collimator and focused onto the sample through a 100× objective lens integrated into a Nikon LV100ND upright microscope. Spatially integrated emission spectra were collected using an Ocean Optics Maya2000 Pro grating spectrometer.

### Scanning Electron Microscopy

A field emission scanning electron microscope (SEM) Zeiss Sigma 300 VP was used to investigate the sample morphology. SEM images were acquired under high-vacuum conditions, variable voltage (within 1 and 5 keV), at a working distance of 3.3–4.5 mm. Samples were imaged with an in-lens detector in top view without any further preparation.

## Supplementary Material



## Notes

Additional data on XRD, PXRD, thin-film XRD, and Raman spectroscopy; DFT calculations, spin-relaxation analysis; temperature-dependent absorption, exciton binding energy analysis, and TRPL; and fluence-dependent ASE, SEM (PDF)
