# Supplementary material for: Structural Rigidity Enables Amplified Spontaneous Emission in 2D Alkylammonium Tin Perovskites
Source: J Am Chem Soc. 2026 Oct 12;148(41):44876–88. doi: 10.1021/jacs.6c14883 (PMC13693382; doi:10.1021/jacs.6c14883)
Supplement: Supplementary file 1 [file ja6c14883_si_001.pdf]

Supporting Information

Structural Rigidity Enables Amplified Spontaneous Emission in 2D Alkylammonium Tin Perovskites

Francesca Canestra,[1]† Nurgul Sarsembek,[1]† Yarong He,[2,3] Andrea Pianetti,[2] Martina Santarelli,[1] Silvia Contessi,[4] Ranita Samanta,[1] Martina Zangari,[1] Federico Brivio,[5] Tommaso Salzillo,[1] Cristina Femoni,[1] Annamaria Petrozza,[2] Daniele Cortecchia[1]*

[1]Department of Industrial Chemistry "Toso Montanari", University of Bologna, 40129, Bologna, Italy
[2]Center for Nano Science and Technology @Polimi, Istituto Italiano di Tecnologia, via Rubattino 81, Milano, 20134 Italy
[3]Department of Physics, Politecnico di Milano, Milano, 20133 Italy
[4]Department of Medical and Surgical Sciences DIMEC, University of Bologna, 40138, Bologna, Italy
[5]Department of Physics and Astronomy "Galileo Galilei", University of Padova, Via Francesco Marzolo 8, 35131, Padova, Italy
† Contributed equally to this work.
** daniele.cortecchia2@unibo.it*

1. X-Ray Diffraction data

Table S1: Structural parameters of the different phases of BA_2SnI_4 and $DABSnI_4$.

**Perovskite**	**125 K BA_2SnI_4 (ref 32)**	**296 K BA_2SnI_4 (ref 32)**	**100 K $DABSnI_4$ (this work)**	**296 K $DABSnI_4$ (this work)**
**Unitary cell**	orthorhombic	orthorhombic	monoclinic	triclinic
**Spatial group**	*P* b c a	*P* b c a	*P* 2_1/c	*P*-1
**a (Å)**	8.43720	8.83700	8.72500	8.46360
**b (Å)**	8.93760	8.61910	8.46250	8.81350
**c (Å)**	26.16700	27.56200	20.44620	11.23160
**α (°)**	90.0000	90.0000	90.0000	77.3510
**β (°)**	90.0000	90.0000	100.8990	68.6770
**γ (°)**	90.0000	90.0000	90.0000	89.8050

Table S2: Crystallographic data for $DABSnI_4$ in both Monoclinic and Triclinic crystal systems.

**Compound**	$DABSnI_4$	$DABSnI_4$
**Empirical Formula**	$[C_4H_{14}N_2]SnI_4$	$[C_4H_{14}N_2]SnI_4$
**Formula weight**	716.46	716.46
**Temperature/K**	100(2)	296(2)
**Crystal system**	Monoclinic	Triclinic

Space group	$P2_1$/c	P-1
**a/Å**	8.7250(3)	8.4636(4)
**b/Å**	8.4625(3)	8.8135(4)
**c/Å**	20.4462(7)	11.2316(5)
**a/°**	90	77.3510(10)
**b/°**	100.8990(10)	68.6770(10)
**g/°**	90	89.8050(10)
**Cell volume/Å^3**	1482.49(9)	758.83(6)
**Z**	4	2
**ρ_{calc}g/cm^3**	3.210	3.136
**m/mm^{-1}**	10.017	9.784
**F(000)**	1256	628
**Crystal size/mm^3**	0.300 × 0.150 × 0.100	0.300 × 0.150 × 0.100
**Radiation**	Mo Kα (λ = 0.71073)	Mo Kα (λ = 0.71073)
**2θ range for data collection/°**	2.029 to 24.999	2.377 to 24.996
**Index ranges**	−10<=h<=10,<br>−10<=k<=10,<br>−24<=l<=24	−10<=h<=10,<br>−10<=k<=10,<br>−13<=l<=13
**Reflections collected**	17853	9530
**Independent reflections**	2610 [R(int) = 0.0432]	2650 [R(int) = 0.0360]
**Completeness to θ max**	100.0 %	98.7 %
**Data/restraints/parameters**	2610 / 0 / 102	2650 / 166 / 158
**Goodness-of-fit on F^2**	1.337	1.177
**Final R indexes [I > 2σ(I)]**	R_1 = 0.0308,<br>wR_2 = 0.0751	R_1 = 0.0348,<br>wR_2 = 0.0783
**R indexes [all data]**	R_1 = 0.0310,<br>wR_2 = 0.0752	R_1 = 0.0382,<br>wR_2 = 0.0799
**Largest diff. peak and hole, e Å^{-3}**	1.893 and −1.057	1.032 and −0.660

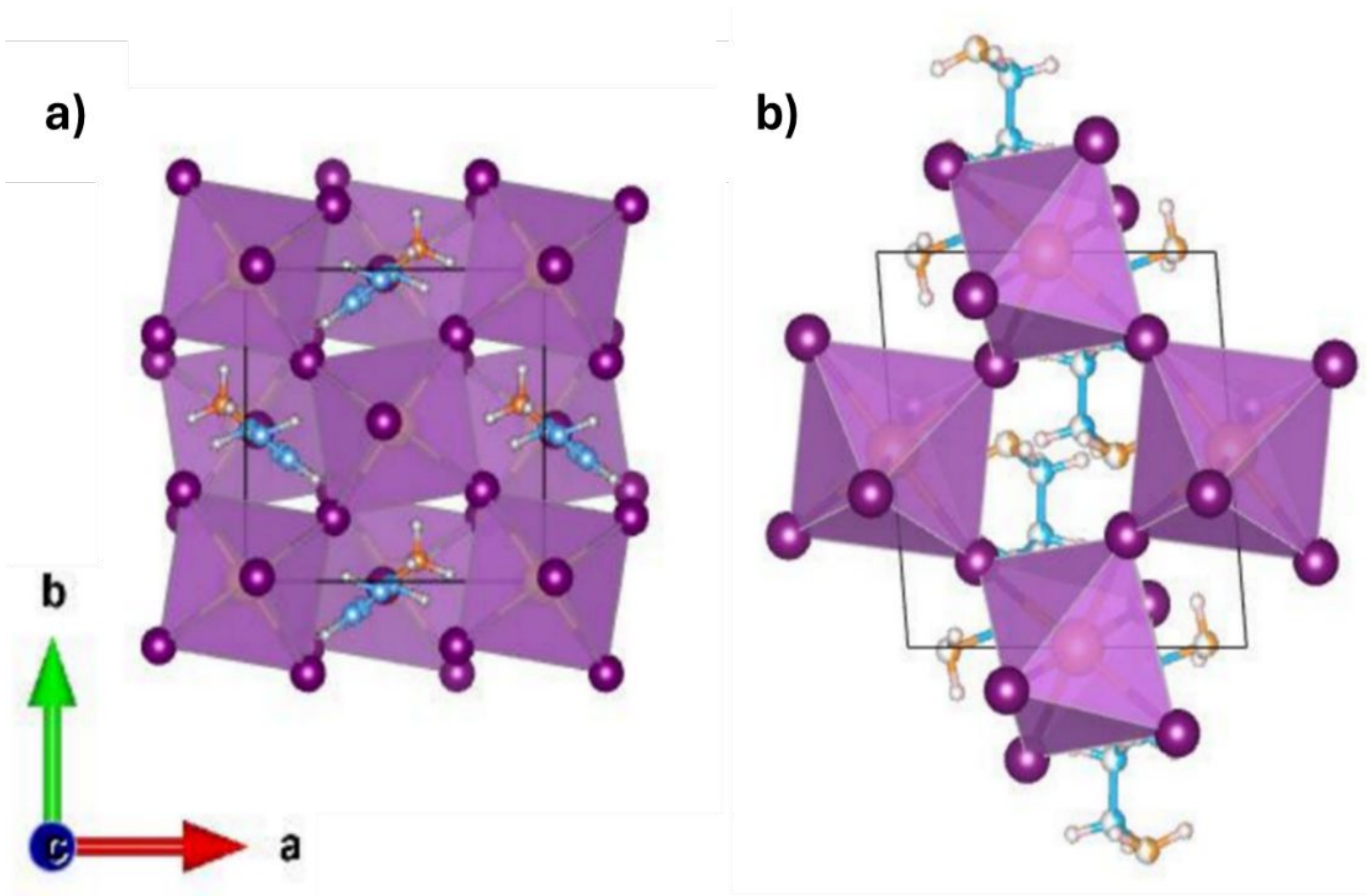

Figure S1: Single crystal at 296 K of the a) BA_2SnI_4 and b) $DABSnI_4$ viewed along the c-axis; a) shows a staggered configuration, confirming the RP phase, while b) shows an eclipsed one, confirming the DJ phase.

Table S3: Changes in the structural parameters after the phase transition, shown in **Figure. 1**. The I-I distance refers to the interlayer distance between iodine atoms caused by the organic spacer and was manually calculated in mercury with the crystal structures. The in-plan angle (D_{in}), the out-of-plane angle (D_{out}) and the octahedral tilting (D_{tilt}), putting as input the coordinates of tin and iodine atoms.[1] The in-plane angle (D_{in}) refers to the angle between two vectors or planes that lie within the same plane, typically describing the spatial relationship between bonds or directions in the 2D lattice structure of a material. On the other hand, the out-of-plane angle (D_{out}) measures the deviation of a vector or plane from the original plane, defined by the angle between the vector or plane and a perpendicular plane. Lastly, tilting (D_{tilt}) describes the distortion of atoms or planes from their ideal or expected arrangement, often involving the rotation or misalignment of structures, such as octahedra, within a crystal lattice.[2] The quadratic elongation quantifies the deviation of bond lengths from the ideal octahedral geometry. It is defined as:

$$\lambda = \frac{1}{4}\sum_{i=1}^{4}\left(\frac{d_i}{d_0}\right)^2 \qquad (1)$$

where d_i are the Sn–I bond lengths, and d_0 is the centre-to-vertex distance of a regular polyhedron with the same volume. The values of the quadratic elongation were derived from VESTA.

Perovskite	Temperature	Interplanar distance (Å)	D_{in}	D_{out}	D_{tilt}	Quadratic elongation
**BA_2SnI_4 (ref.32)**	125 K	6.93	20	21	28	1.0014
	296 K	7.50	19	6	20	1.0014
**$DABSnI_4$ (this work)**	100 K	3.90	22	20	30	1.0009
	296 K	4.02	22	20	30	1.0011

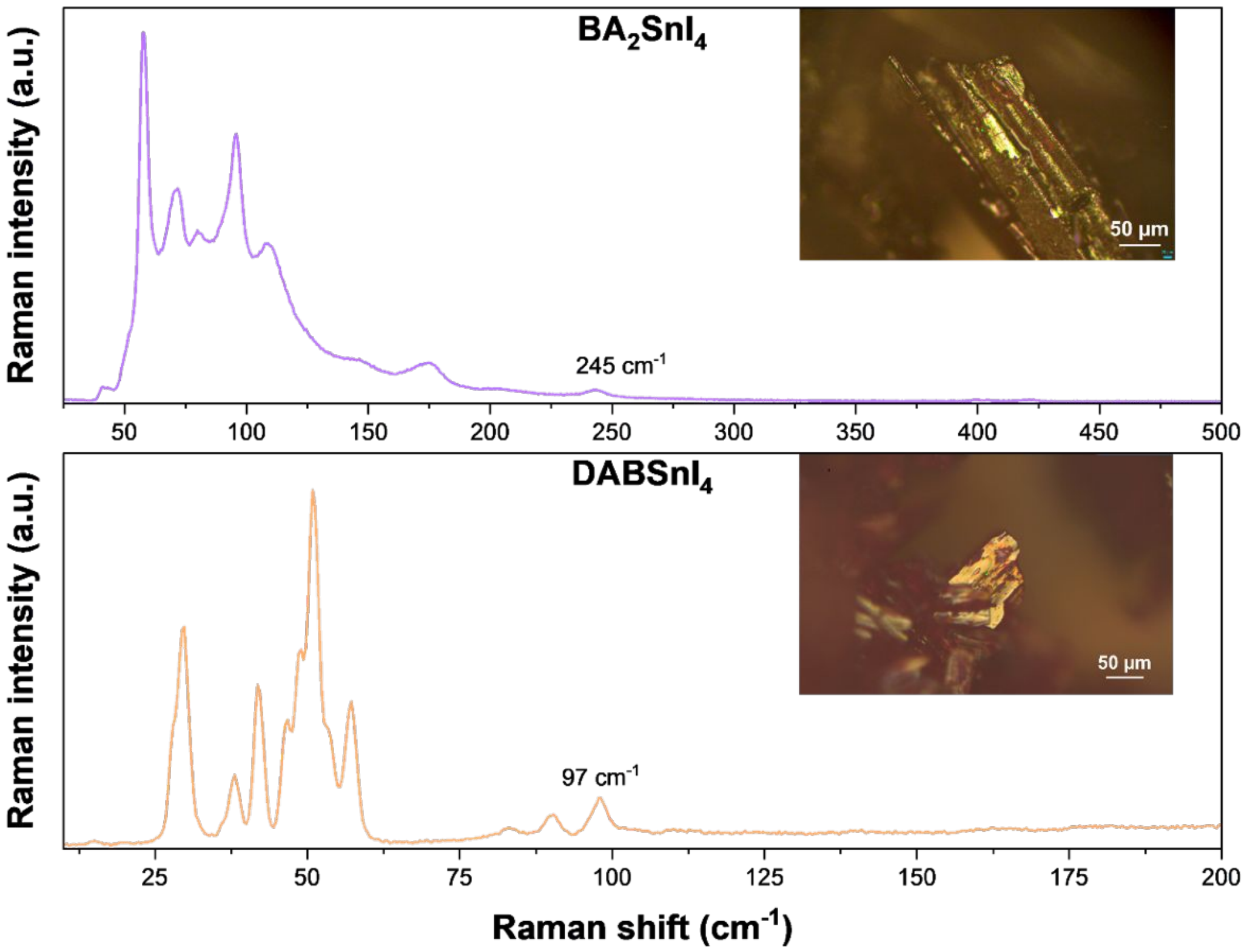

Figure S2: Raman spectroscopy of the inorganic octahedra of BA_2SnI_4 and $DABSnI_4$ at 78 K, measured with a 647 nm laser and a 785 nm laser, respectively. The wavenumbers in figures correspond to the exciton-phonon coupling mode.

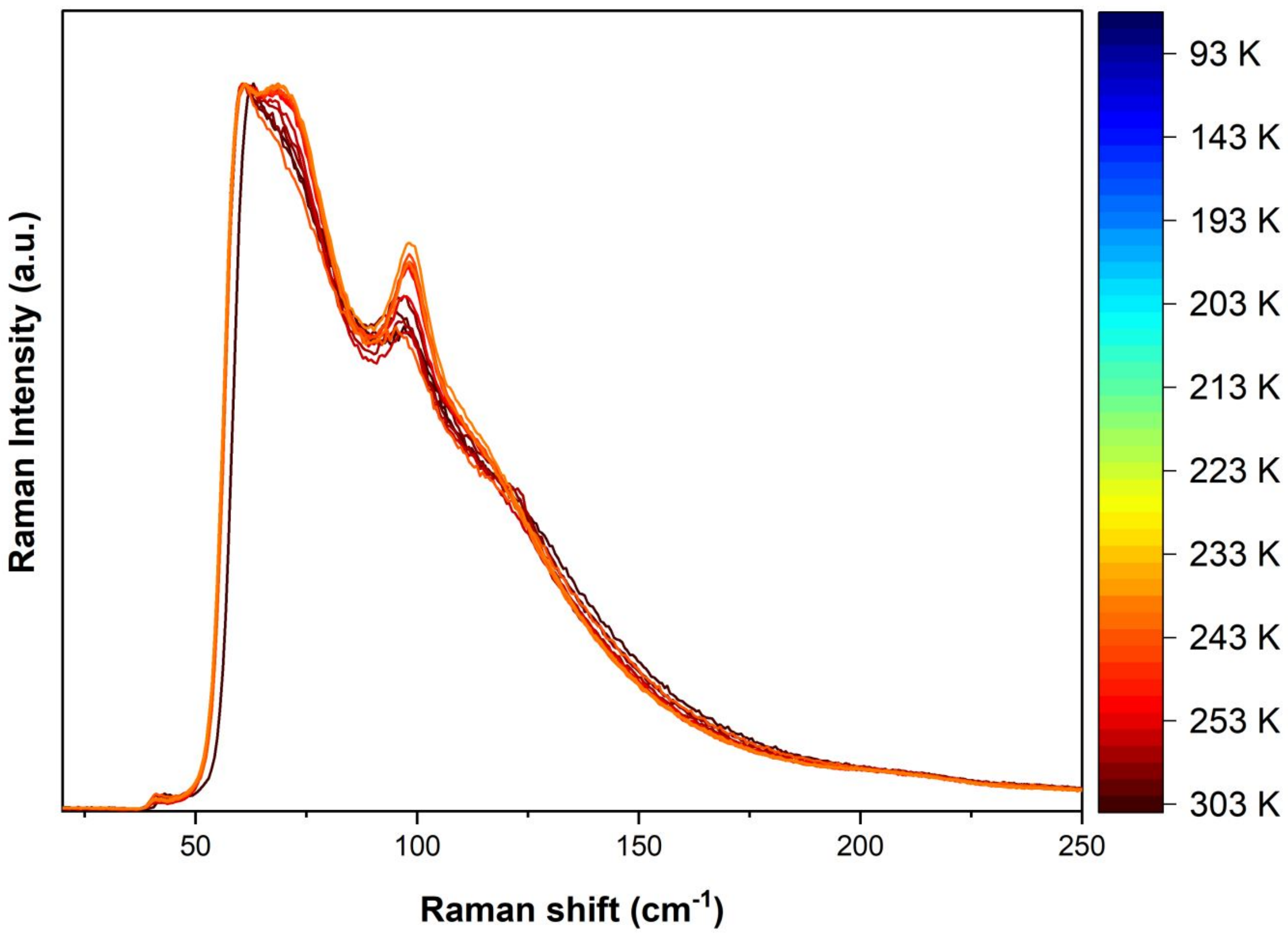

Figure S3: Raman spectra of the high temperature phase of BA_2SnI_4 (shown in **Fig. 1f**) from 239 K to 303 K.

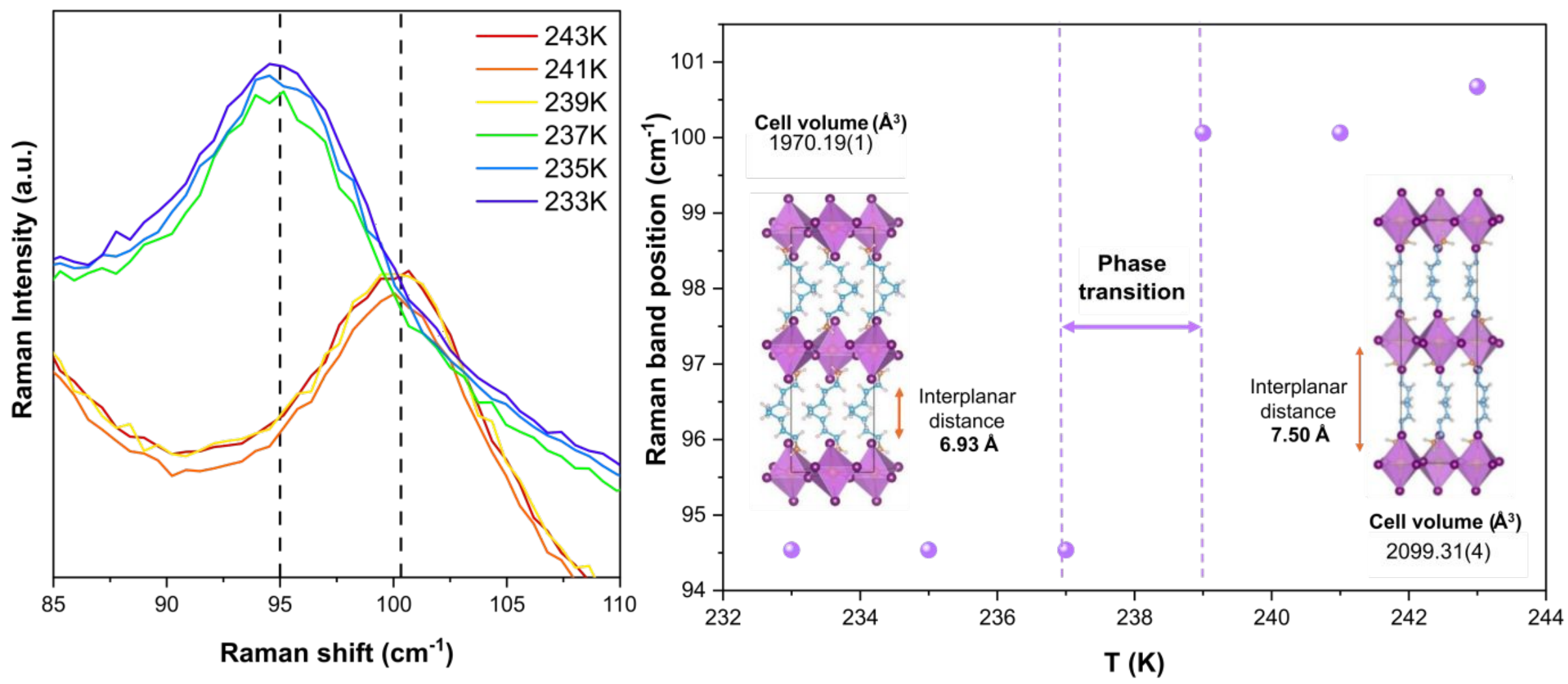

Figure S4: a) Detail of the Raman mode associated to libration modes of the organic cations coupled to Sn–I stretching, which particularly affected by the phase transition. b) Evolution of the Raman peak center as a function of temperature, highlighting the changes in peak position upon the phase transition. The Raman mode is associated with a change in the cell volume and interplanar distance between the low-temperature structure at 125 K, before the transition, and the room-temperature (296 K) structure, after the transition.

Prior to the ssNMR experiments, we confirmed that grinding the crystals did not affect their crystalline structure. The X-ray powder diffraction pattern of the ground sample was identical to that of the pristine crystal, confirming structural integrity after grinding (see **Figure. S5**).

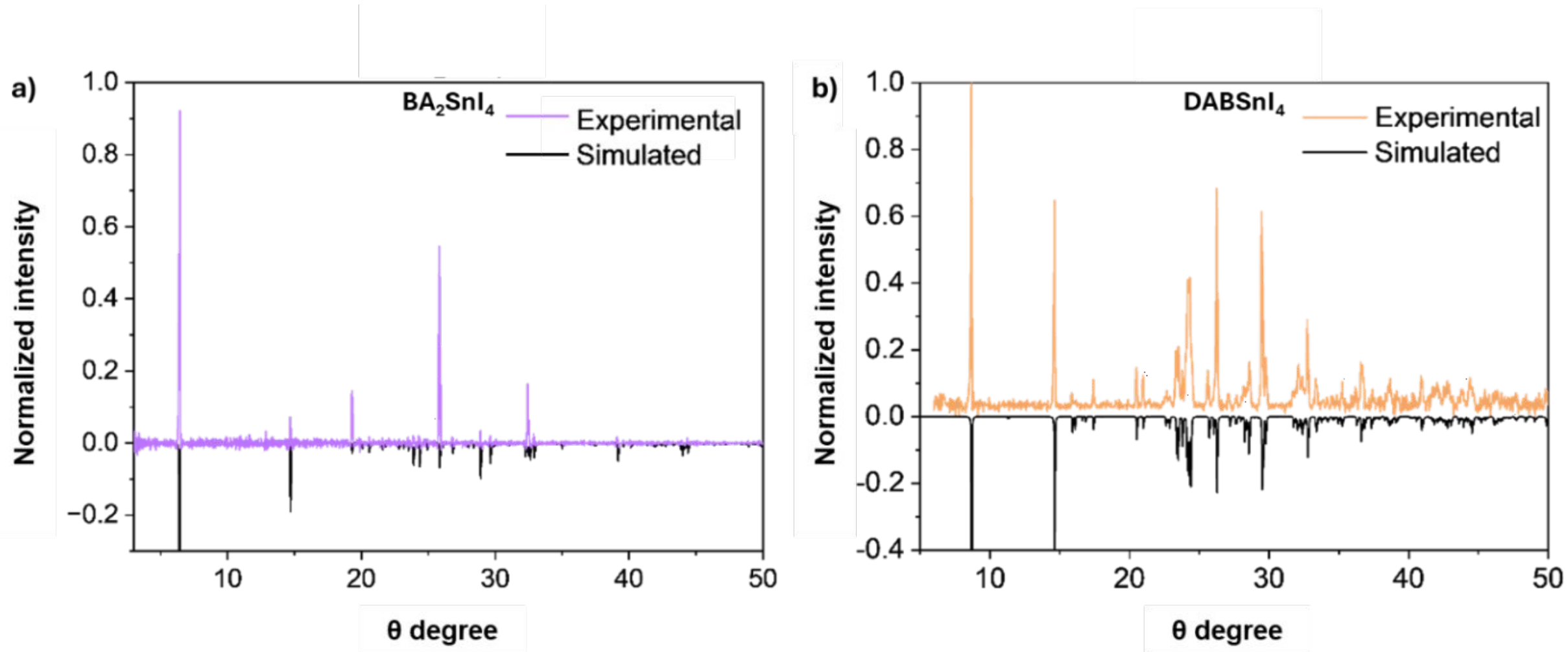

Figure S5: Comparison of the experimental and simulated powder x-ray diffraction patterns of the perovskites. In both cases the experimental peaks correspond with the simulated ones. In the case of BA_2SnI_4 the most intense peaks in the simulated diffractogram positioned at 6.4°, 14.7°, and 8.9° correspond to experimental pattern, and are associated with the reflections from (002), (111), and (220) crystal planes, respectively. Also, for $DABSnI_4$ we observe a good correspondence both in terms of position and relative intensity. Examples of this are the strongest signals at 8.7° (001), 14.6° (111), 26.3° (003), 29.5° (222), 32.8° (132).

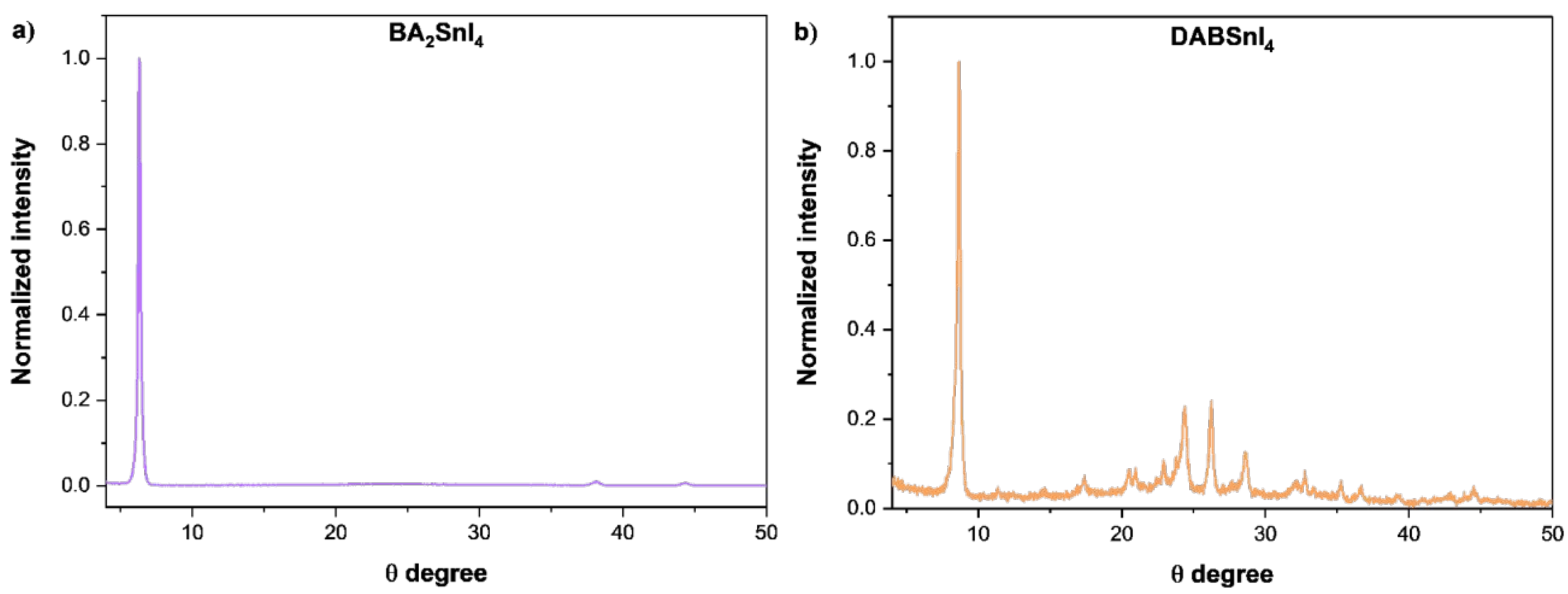

Figure S6: Normalized thin film XRD patterns of BA_2SnI_4 (a) and $DABSnI_4$ (b) films. BA_2SnI_4 shows a single major peak at 6.3°, indicating preferential orientation along the (002) plane and the formation of an ordered, compact polycrystalline structure. $DABSnI_4$ films display a dominant peak at 8.6° followed by several diffraction peaks in the range 15–45°, indicating a lower tendency to preferentially orient the perovskite layers parallel to the substrate.

2. Supporting ssNMR

2.1. CP-MAS $^{13}C\ \{^1H\}$ ssNMR signal build-up for BA_2SnI_4

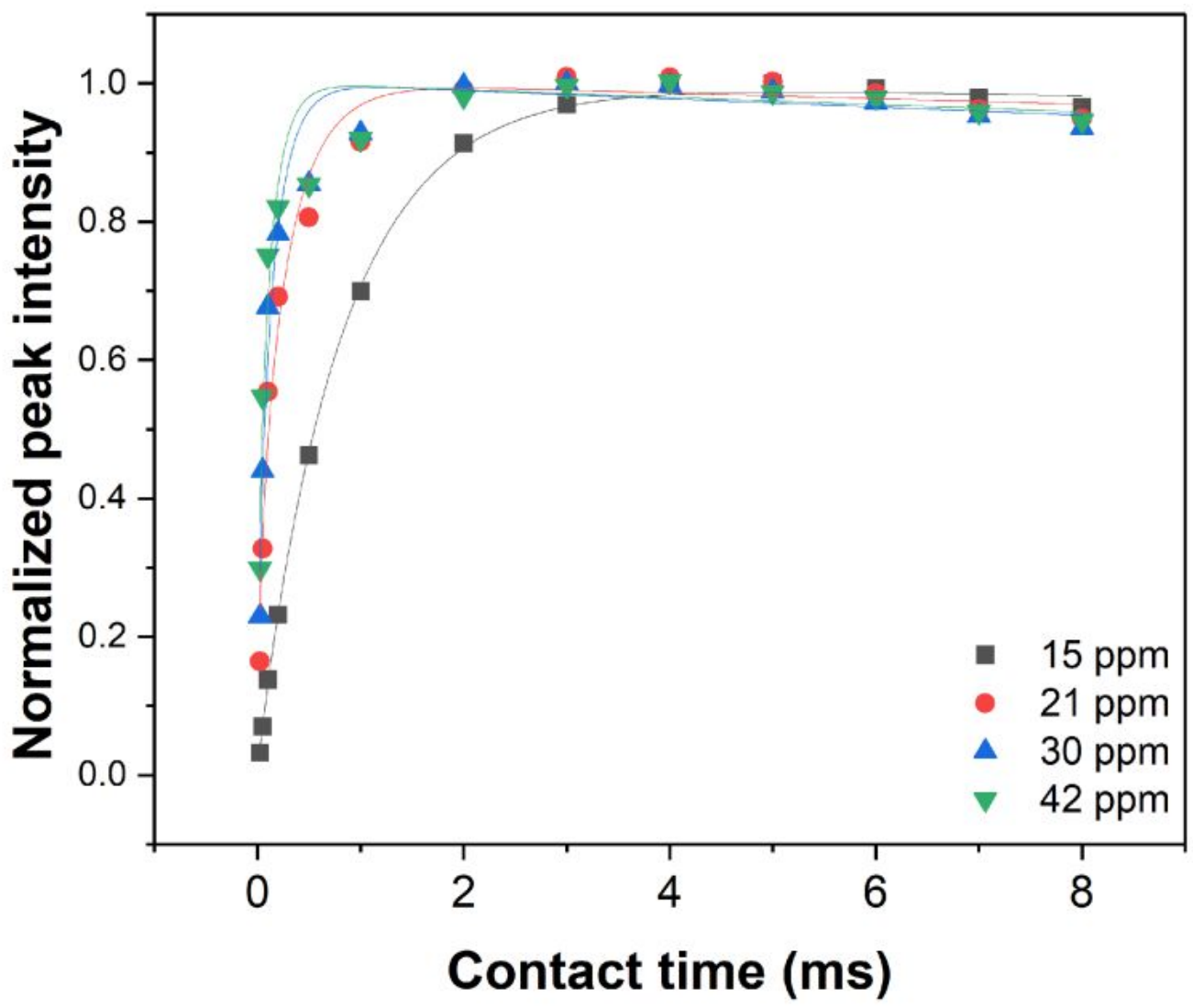

Figure S7: a) CP-MAS $^{13}C\ \{^1H\}$ ssNMR signal build-up curves for four distinct carbon sites in the BA cation (symbols), measured at *5* kHz MAS, with corresponding monoexponential fitting curves. The data represent the $^1H \rightarrow {}^{13}C$ magnetization transfer according to the equation: $I(t) = I_0(1 - e^{-Kcp})$, where I(t) is the signal intensity at a given contact time t for each individual carbon, I_0 is the maximum intensity, and Kcp is the cross polarization transfer rate, which depends on dipole-dipole interactions between the nuclei.

2.2. Chemical Shielding and Chemical Shift from DFT calculations for $DABSnI_4$

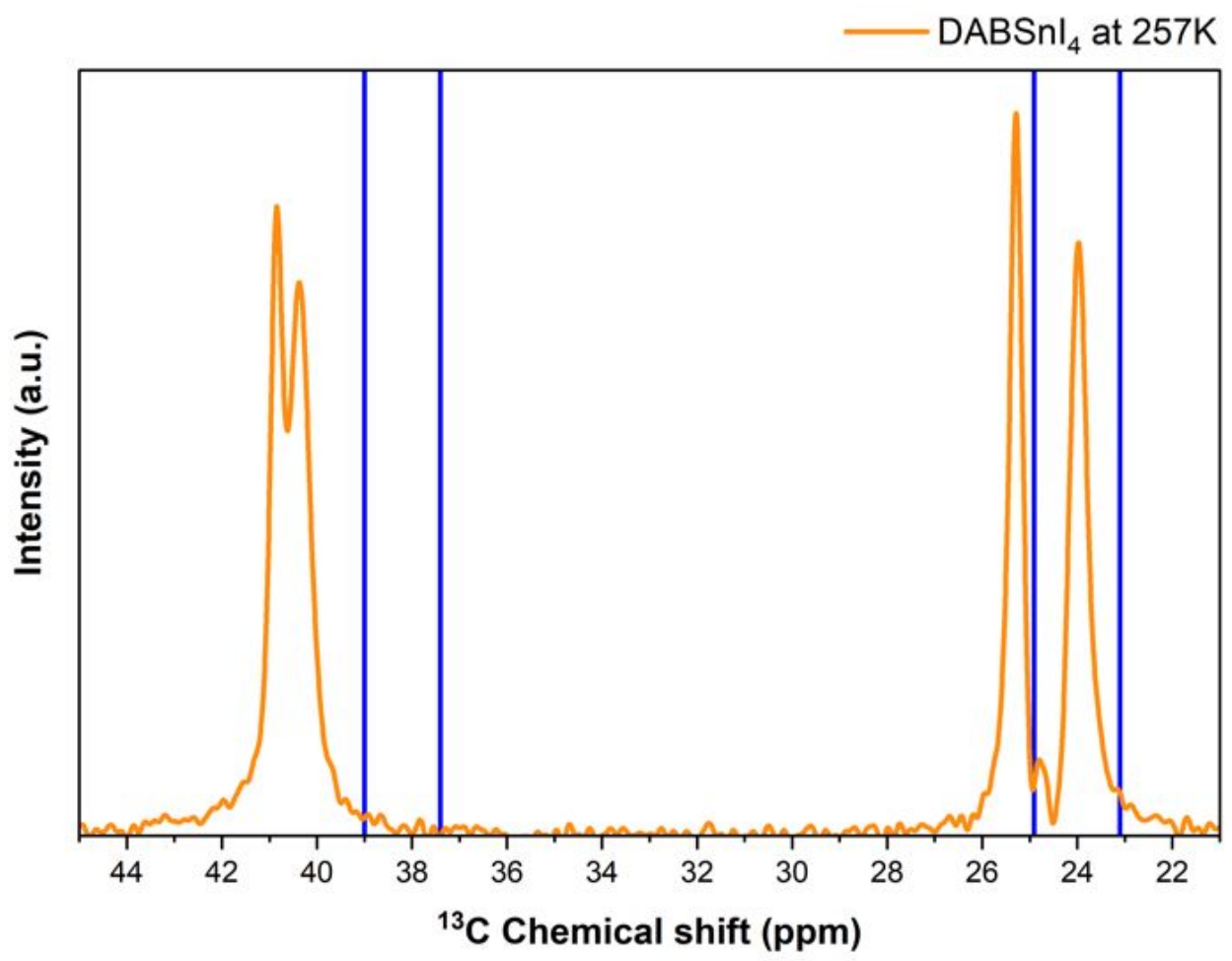

Figure S8: Comparison between experimental data of $DABSnI_4$ at 275 K (orange line) and NMR spectra simulated with DFT on equilibrium structure (vertical blue lines). DFT data do not include intensity and just the peak position is reported. Computational data well reproduces

the experimental data with a small (< 3 ppm) systematic error that is within the expectation of the method used. The peak positions are also reported in **Table S4.**

Table S4: Tabulated value for the DFT calculations of $DABSnI_4$ reported in **Figure S8.**

**Calculated ^{13}C Chemical shifts (ppm)**
Temperature = 100 K, ppm
37.4
24.9
23.1
39.0

NMR experiment data are usually reported in terms of chemical shift, since the chemical shielding of the material is reported with respect to a known sample. Since DFT simulation allows for the direct calculation of the chemical shielding, the comparison with experiment requires an analogous *in silico* process to obtain the chemical shift. This is done using a linear fit of both experimental chemical shift and computational obtained chemical shielding of known compound. In our case we took this compound from this publication.[3] This reduces the influence of possible systematic errors.

For a given atomic species i :

$$\sigma_i = c_1 + c_2\,\delta_i$$

where σ_i is the chemical shift of the atomic species i , δ_i the chemical shielding obtained by calculation. The parameters c_1 and c_2 are obtained via linear least-squares fit between experimental and DFT data of a chosen dataset. In an ideal case the first parameter is the chemical shielding of the reference material used in the experiment and the second parameter is –1.

In this study, as in our previous paper,[4] we have taken the dataset shown in **Table S5** from the work of Hartman et. al,[3] and we found $c_1 = 173.7\ and\ c_2 = -1.020,\ R^2 = 0.9976$.

Table S5: Experimental chemical shift δ and theoretical chemical shielding σ used to obtain the ^{13}C chemical shift for our systems.

Name	CSH [ppm]	CSF [ppm]	Name	CSH [ppm]	CSF [ppm]	Name	CSH [ppm]	CSF [ppm]
**ADENOS12**	155	18	**INDMET**	179	12	**LTYROS11**	176	-7
	155	21		168	2		156	13
	149	27		157	13		131	39
	138	35		140	26		130	40
	120	51		138	32		123	50
	92	76		137	38		117	52
	85	85		134	38		117	54
	75	95		132	38		55	118
	71	99		131	40		36	135
	63	109		131	40	**MEMANP11**	100	68
**ASPRM03**	177	-3		129	42		72	99
	176	-9		127	44		72	100
	52	125		116	56		71	101
	36	138		113	57		65	108
**FRUCTO02**	100	68		113	61	**MGALPY01**	59	114
	71	99		98	77		55	116
	69	104		55	117		100	68
	67	105		28	146		73	98
	65	105	**LALNIN12**	14	161		73	99
	65	106		177	-9		70	105
**GLTUAM01**	177	-4		51	124		68	110
	174	-4		20	154		61	117
	54	122	**LCYSTN21**	175	-4		55	120
	29	145		54	119	**MGLUCP11**	101	67
	26	148		35	142		75	97
**HXCAN09**	170	4	**LSERIN01**	175	-7		75	97
	152	19		63	106		73	99
	133	40		56	119		72	101
	123	47	**LSERMH10**	176	-8		63.8	108
	121	51		62	109		57	114
	116	55		58	116	**NAPHTA36**	135	38
	116	56	**LTHREO01**	155	19		130	39
	24	149		114	60		129	40
				104	65		126	44
				-2	170		125	45

2.3. Spin-relaxation analysis

The temperature dependence of the ^1H spin–lattice relaxation times (T_1) was analyzed using the Bloembergen–Purcell–Pound (BPP) model,[5] which describes nuclear spin relaxation driven by dipole–dipole interactions modulated by molecular motions. In this model, the relaxation rate is expressed as:

$$\frac{1}{T_1} = C\left[\frac{\tau_c}{1+\omega_0{}^2\tau_c{}^2} + \frac{2\tau_c}{1+4\omega_0{}^2\tau_c{}^2}\right] \quad (1)$$

where ω_0 is the Larmor frequency and τ_c represents the correlation time of motion. The prefactor C depends on the strength of the dipolar interaction and is defined as:

$$C = \frac{3m_0^2\hbar^2g^4}{160p^2r^6} \quad (2)$$

with μ_0 is the vacuum permeability, ħ is the reduced Planck constant, γ is the gyromagnetic ratio of the proton, and r is the internuclear separation between coupled protons.
The temperature dependence of the correlation time was modelled assuming thermally activated motions following Arrhenius equation:[6]

$$\tau_c = \tau_0 * e^{\frac{Ea}{RT}} \quad (3)$$

where τ_0 is a pre-exponential factor, T is the temperature, R is the gas constant and Ea is the activation energy. The experimental T_1 data were fitted using equations (1) and (3), and corresponding activation parameters were extracted from the fits shown in **Figure 3d** and summarized in **Table S6.**

Table S6: Fitting parameters of temperature-dependent ^1H T_1 measurements for BA_2SnI_4 and $DABSnI_4$.

Sample	τ_0 (ps)	Ea (kJ/mol)
BA_2SnI_4	0.26 ± 0.46	7.71 ± 2.82
$DABSnI_4$	0.17 ± 0.11	13.94 ± 1.97

3. Temperature dependent optical characterization

3.1. Absorption spectra

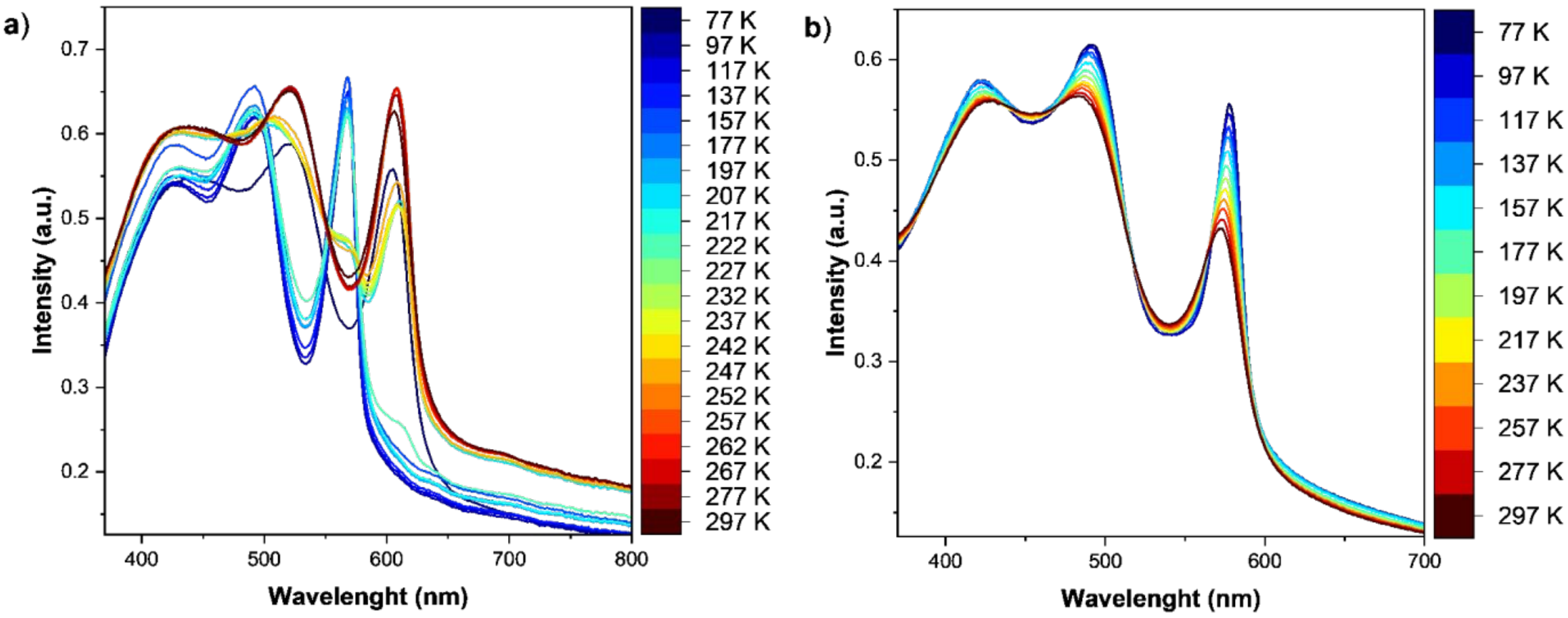

Figure S9: Absorption spectrum at different temperatures of a) BA_2SnI_4, where the phase transition can be clearly seen and b) of $DABSnI_4$, where there is not a marked discontinuity in the temperature dependence spectra evolution also across the phase transition, in agreement with the structural parameters.

3.2. Photoluminescence analysis

The steady state photoluminescence spectra were deconvoluted in excitonic and trap emission. In BA_2SnI_4 it was necessary to consider two contributions to fit the line shape of the excitonic emission. This is a similar case to the fine structure splitting of the exciton manifold observed in PEA_2SnI_4 and other 2D perovskites (although with a much less marked spectral separation of the individual contributions),[7,8] which has previously been attributed to the formation of exciton-polarons.[9–11] The two independent contributions, indicated as exciton 1 and exciton 2 in **Figure S10**, needs to be taken into account for the low temperature phase of BA_2SnI_4, while they cannot be individually distinguished after phase transition beyond 250 K due to the line broadening at high temperature (**Figure S11**).

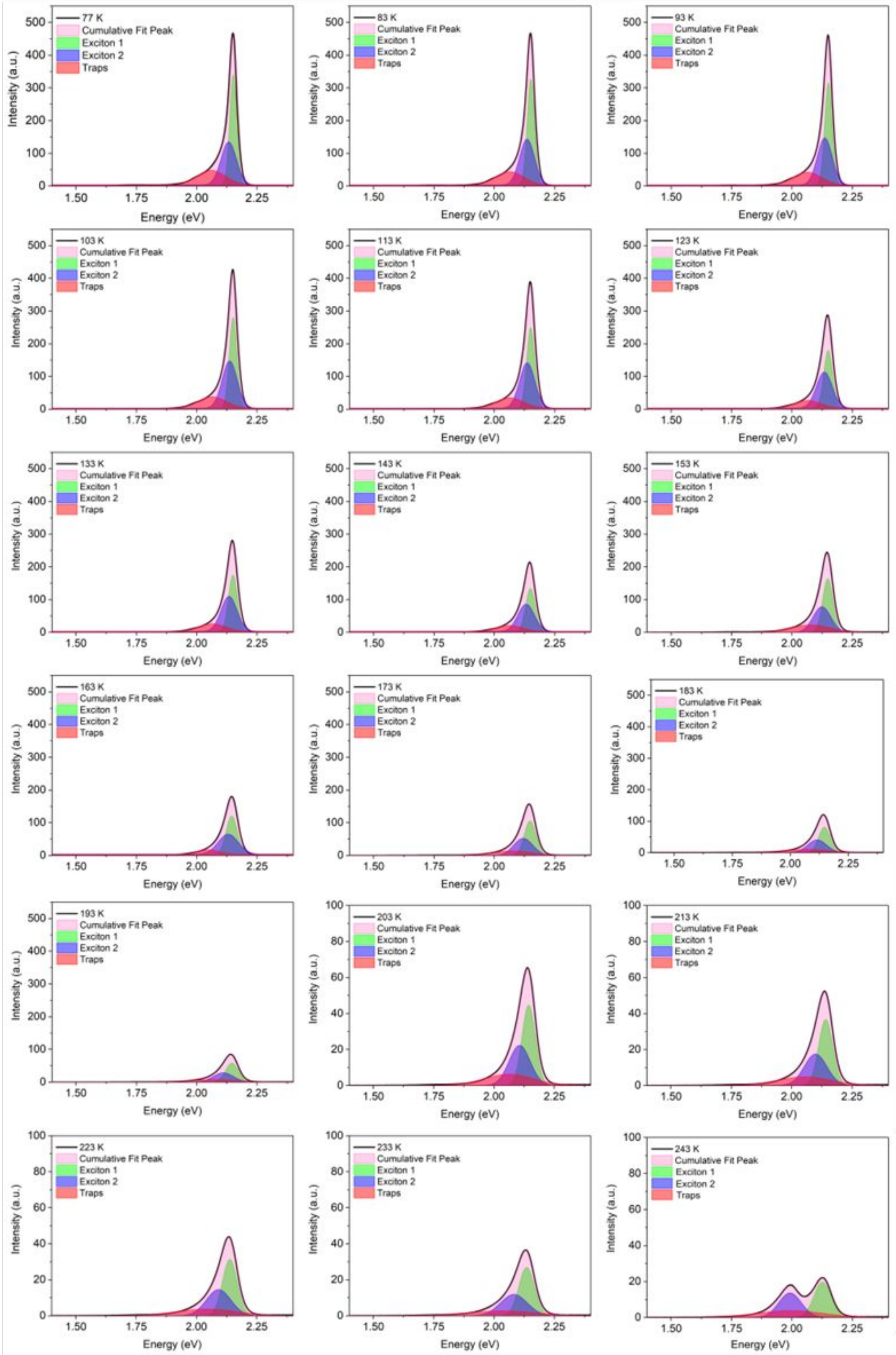

Figure S10: Fitting of the PL peaks of BA_2SnI_4. These fittings are done in the low temperature phase (before the phase transition, from 77 K to 243 K), using 3 peaks: two excitonic transitions indicated as exciton 1 and 2 (green at 2.15 eV and blue at 2.13 eV) and one low-energy band related to trap states (red at 2.06 eV).

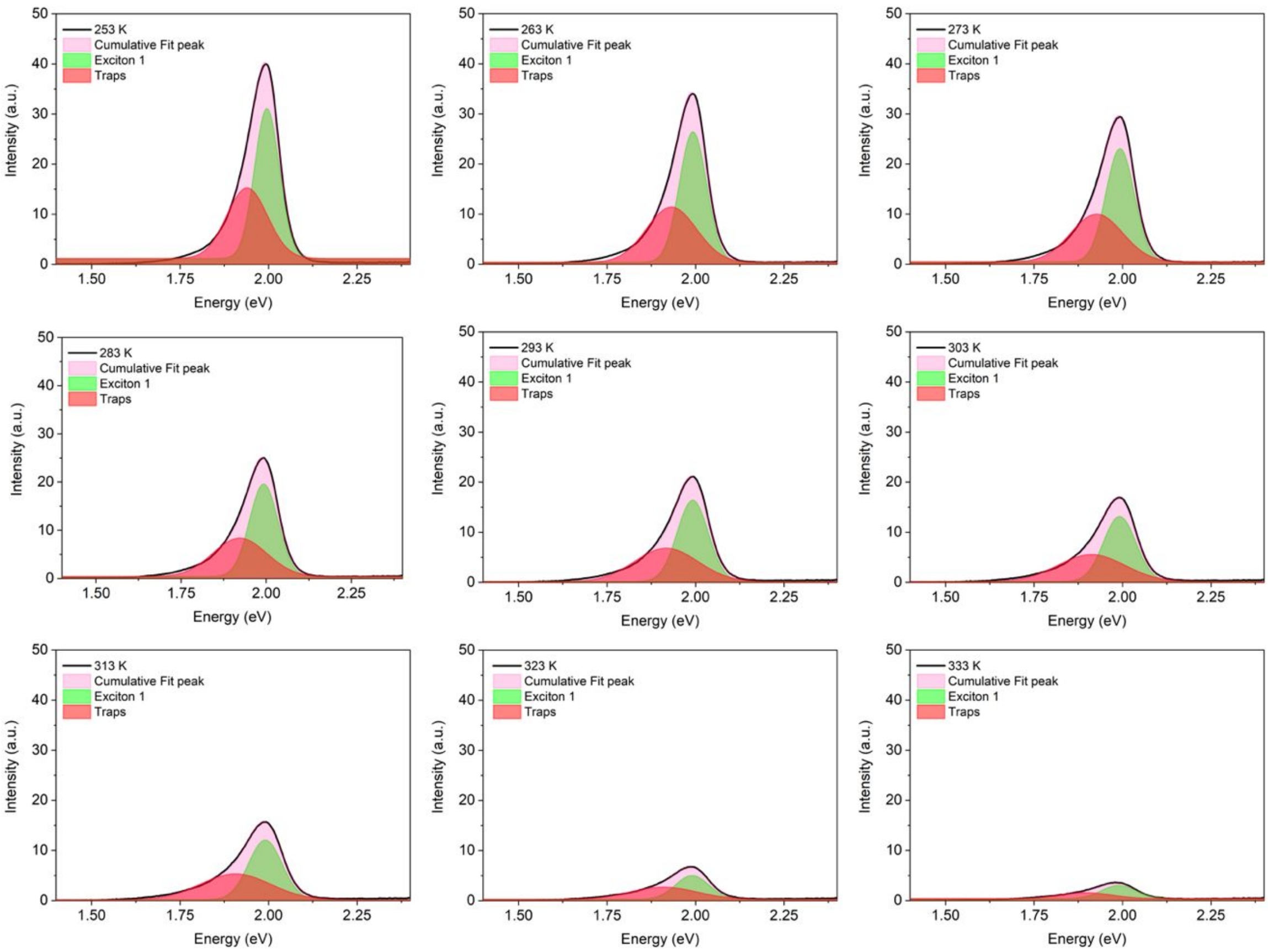

Figure S11: Fitting of the PL peaks of BA_2SnI_4. These fittings are done in the high temperature phase (after the phase transition, from 253 K up to 333 K), using 2 peaks: one excitonic, called exciton 1 (green at 2.02 eV) and one band related to trap states (red at 1.92 eV).

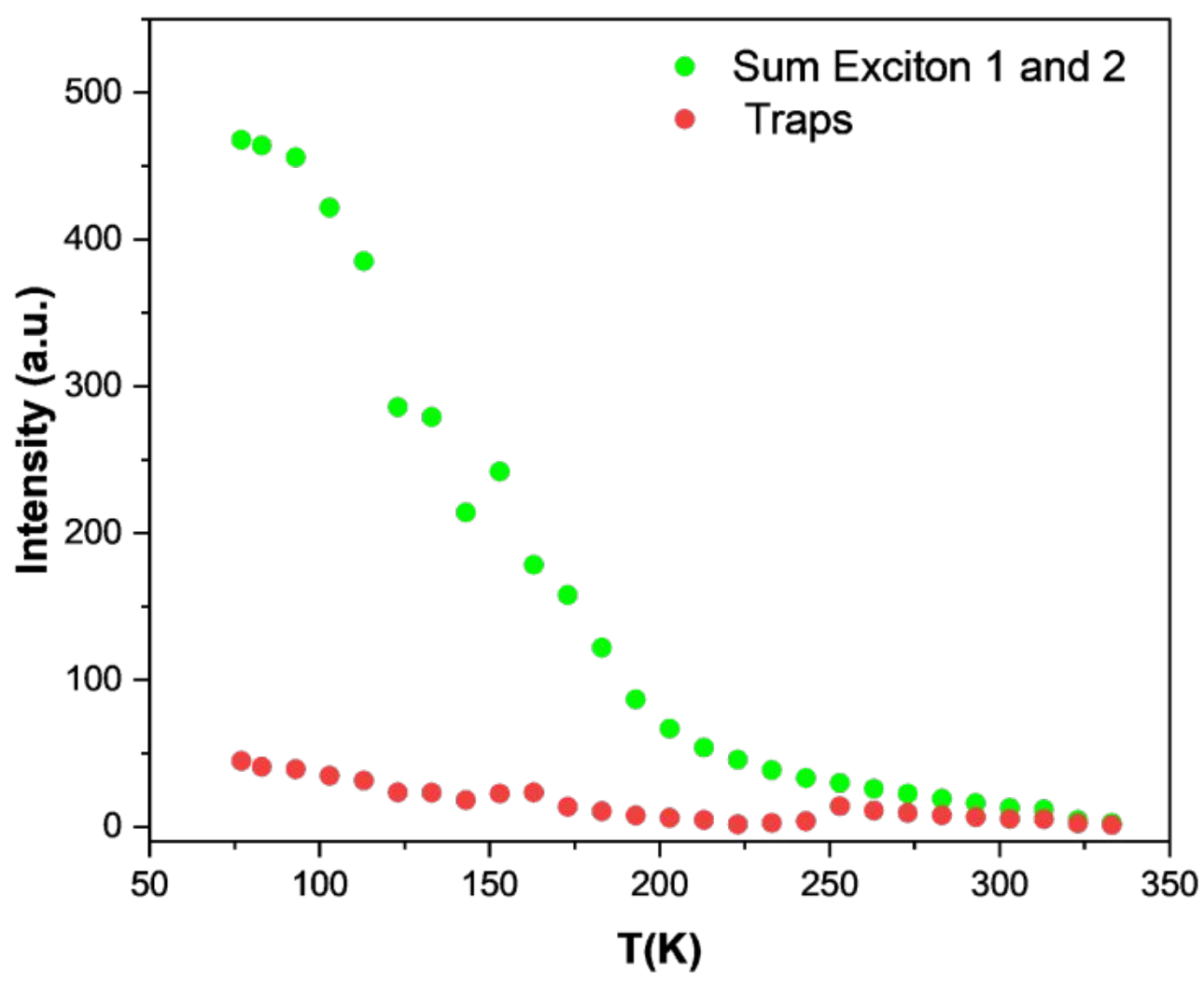

Figure S12: Evolution of the excitonic and traps contribution with temperature for BA_2SnI_4. In green there's the sum of the Exciton 1 and Exciton 2 intensities (see **Figure. S10,11**), while in red the traps one.

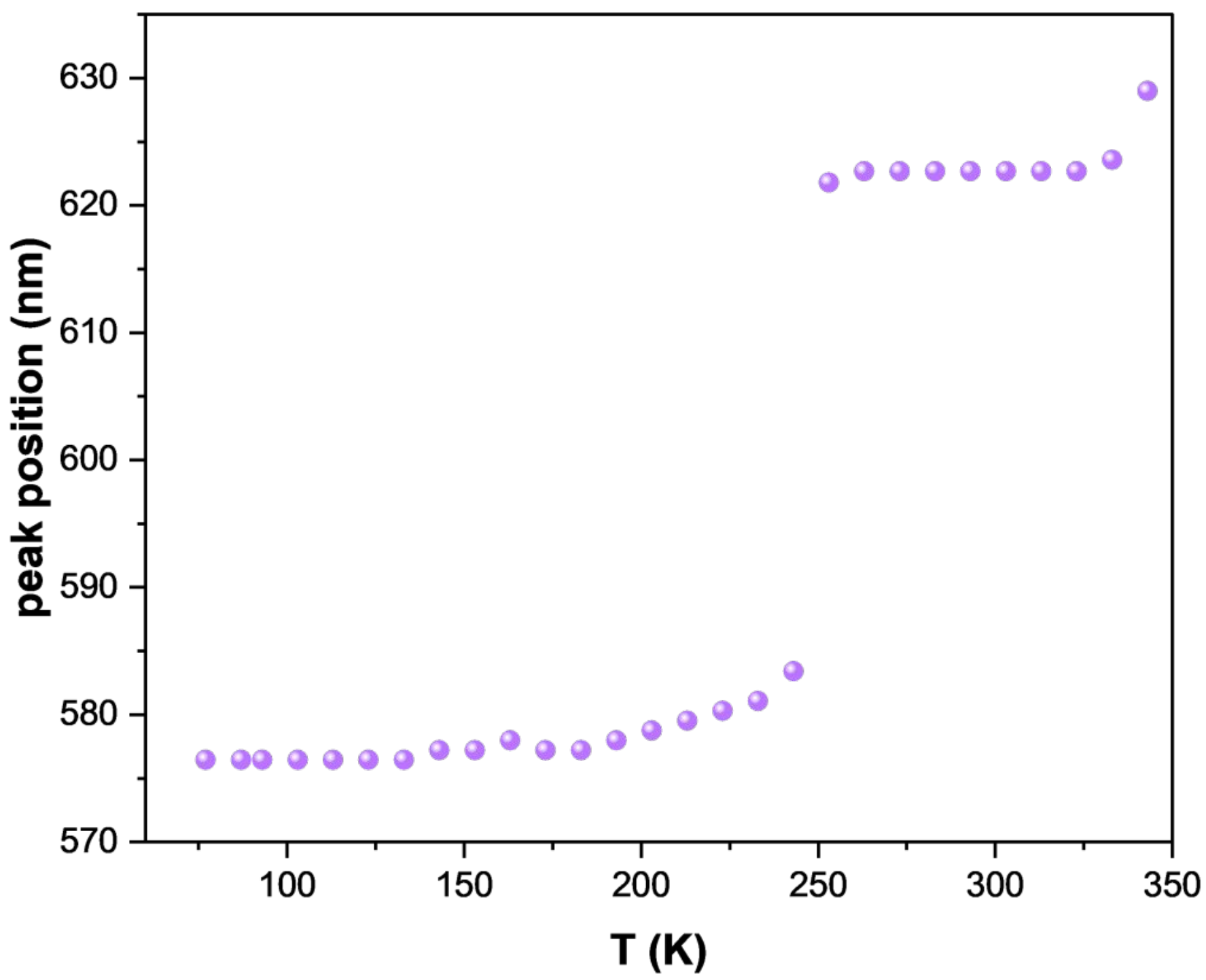

Figure S13: Temperature dependence of the excitonic peak position, obtained by determining the peak center of the PL spectra shown in Fig. 4b. A sudden shift of approximately 40 nm is observed at 243 K, corresponding to the phase transition.

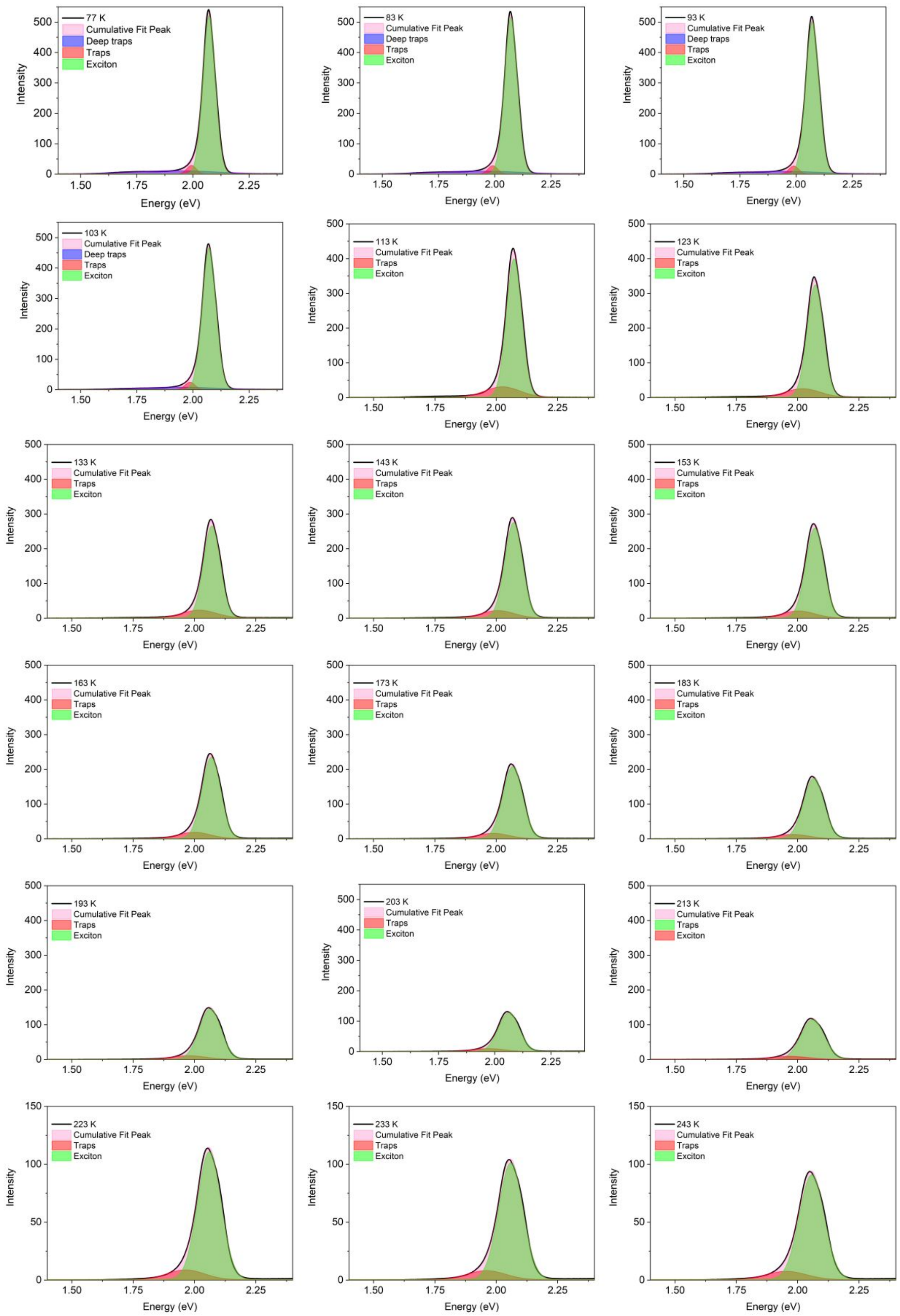

Figure S14: Fitting of the PL peaks of $DABSnI_4$. The fittings showed in picture are from 77 K up to 243K, using two peaks for temperatures between 113-243 K: one excitonic band (green at 2.07 eV) and one band related to trap states (red). For lower temperatures (77 K −103 K), a weak highly Stokes-shifted broadband emission related to deep trap states was further identified (blue peak at 700 nm), with a marginal contribution compared to the main excitonic emission.

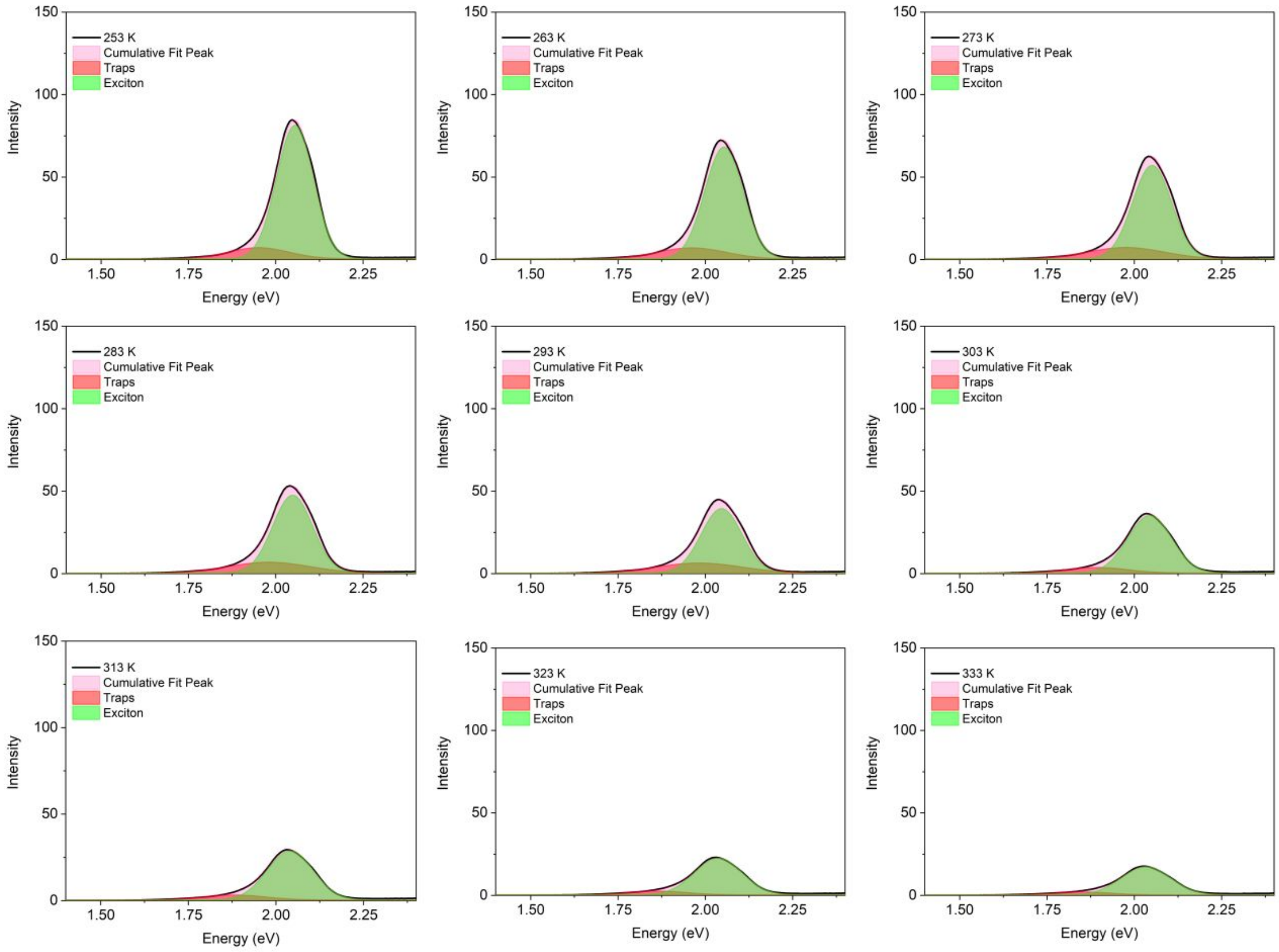

Figure S15: Fitting of the PL peaks of $DABSnI_4$. These fittings shown in picture are from 253 K up to 333 K, using two contributions: one excitonic band (green at 2.07 eV) and one band related to trap states (red).

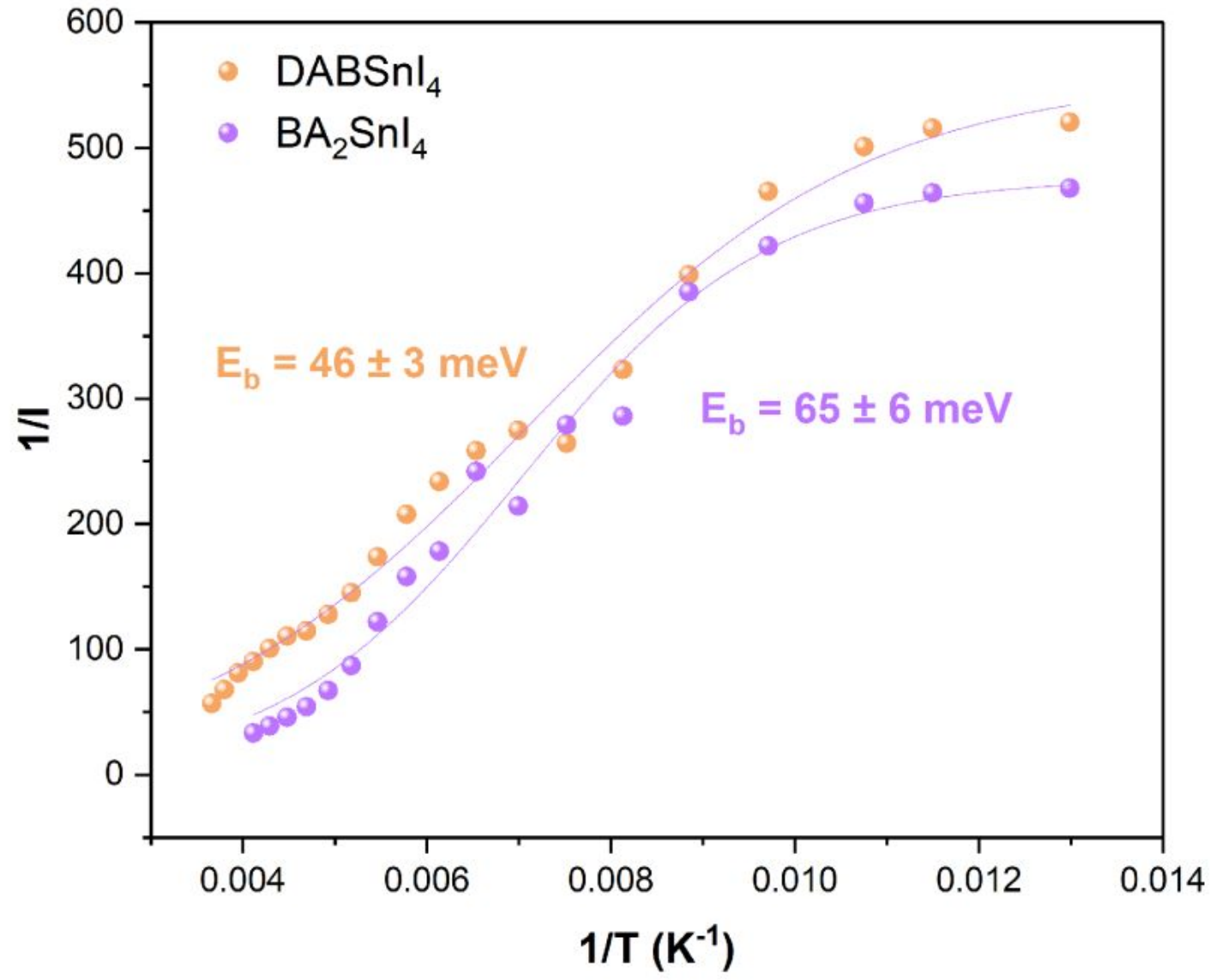

Figure S16: Fitting of the intensity vs temperature of the perovskite low temperature phase (before phase transition) with the Arrhenius plot. The integrated PL intensity as a function of temperature is used to derive the exciton binding energy (E_b) using the formula:

$$I(T) = \frac{I_0}{1 + Ae^{(\frac{-E_b}{k_B T})}} \quad (2)$$

where I(T) is the integrated PL intensity at temperature T, A is a constant, k_B is the Boltzmann constant, and E_b is the exciton binding energy. For BA_2SnI_4 the intensity is represented by the sum of the two excitonic contributions (the ones at 2.15 eV and 2.13 eV).

Table S7: Results of the fitting of the PL in temperature: The exciton–phonon coupling strength in these materials was evaluated through the temperature-dependent broadening of the PL spectra, described by **Equation 1** where, Γ_{inh} is the inhomogeneous peak width at zero temperature, Γ_{LO} is the exciton-longitudinal optical (LO) phonon coupling coefficient, k_B is the Boltzmann constant, and E_{LO} is the LO phonon energy.
The integrated PL intensity as a function of temperature is used to derive the exciton binding energy (E_b) using the formula, indicated in **Equation 2**, where I(T) is the integrated PL intensity at temperature T, A is a constant, k_B is the Boltzmann constant, and E_b is the exciton binding energy. The uncertainties on the fitted parameters correspond to the standard errors returned by the nonlinear least-squares fitting routine implemented in Origin, calculated from the covariance matrix of the fit parameters.

Sample		Γ_{inh} (meV)	Γ_{LO} (meV)	E_{LO} (meV)	E_b (meV)
BA_2SnI_4	T < 243 K	32 ± 4	175 ± 29	32	65 ± 6
	T > 243 K	38 ± 5	163 ± 14	32	/
$DABSnI_4$	77 K – 333 K	53 ± 8	57 ± 9	12	46 ± 3

Time Resolved Photoluminescence

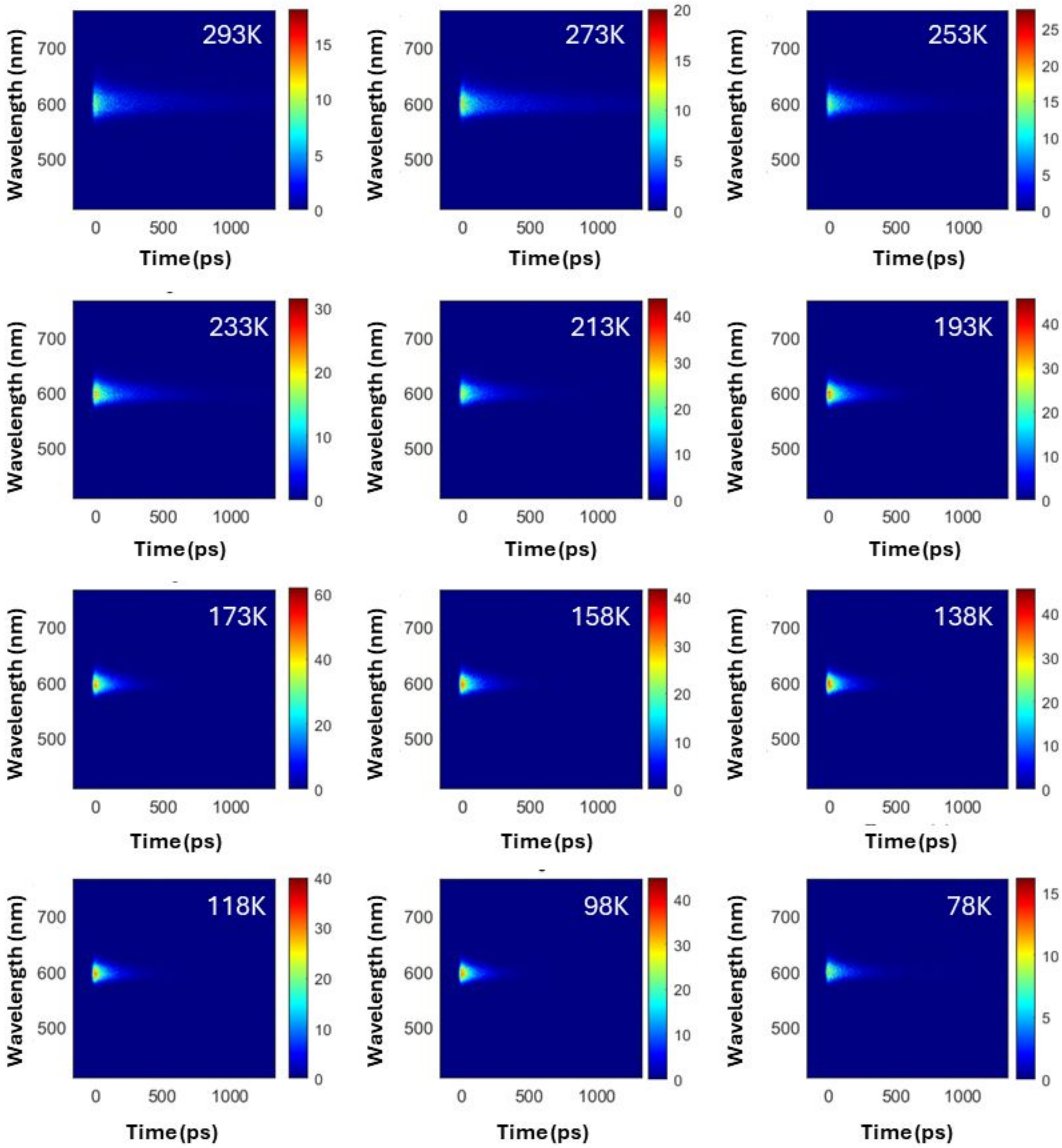

Figure S17: Time Resolved Photoluminescence at different temperatures of $DABSnI_4$, performed with an excitation wavelength of 515 nm, 80 MHz, Power out 0.55 µW (0.120 on samples), with a grating of 50 gr/mm

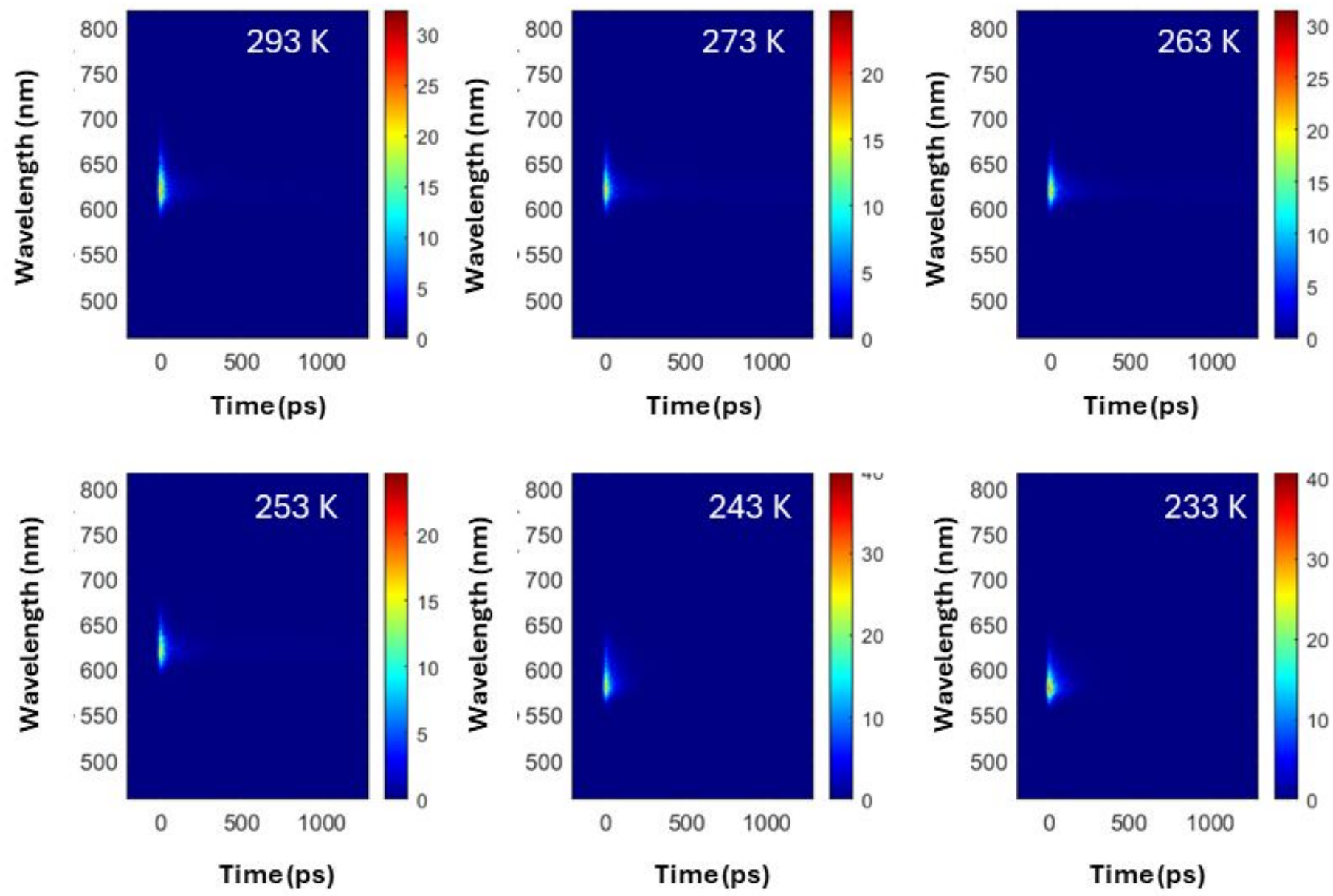

Figure S18: Time Resolved Photoluminescence at different temperatures of BA_2SnI_4, performed with an excitation wavelength of 515 nm, 80 MHz, Power out 1.55 µW (0.140 on samples), with a grating of 150 gr/mm

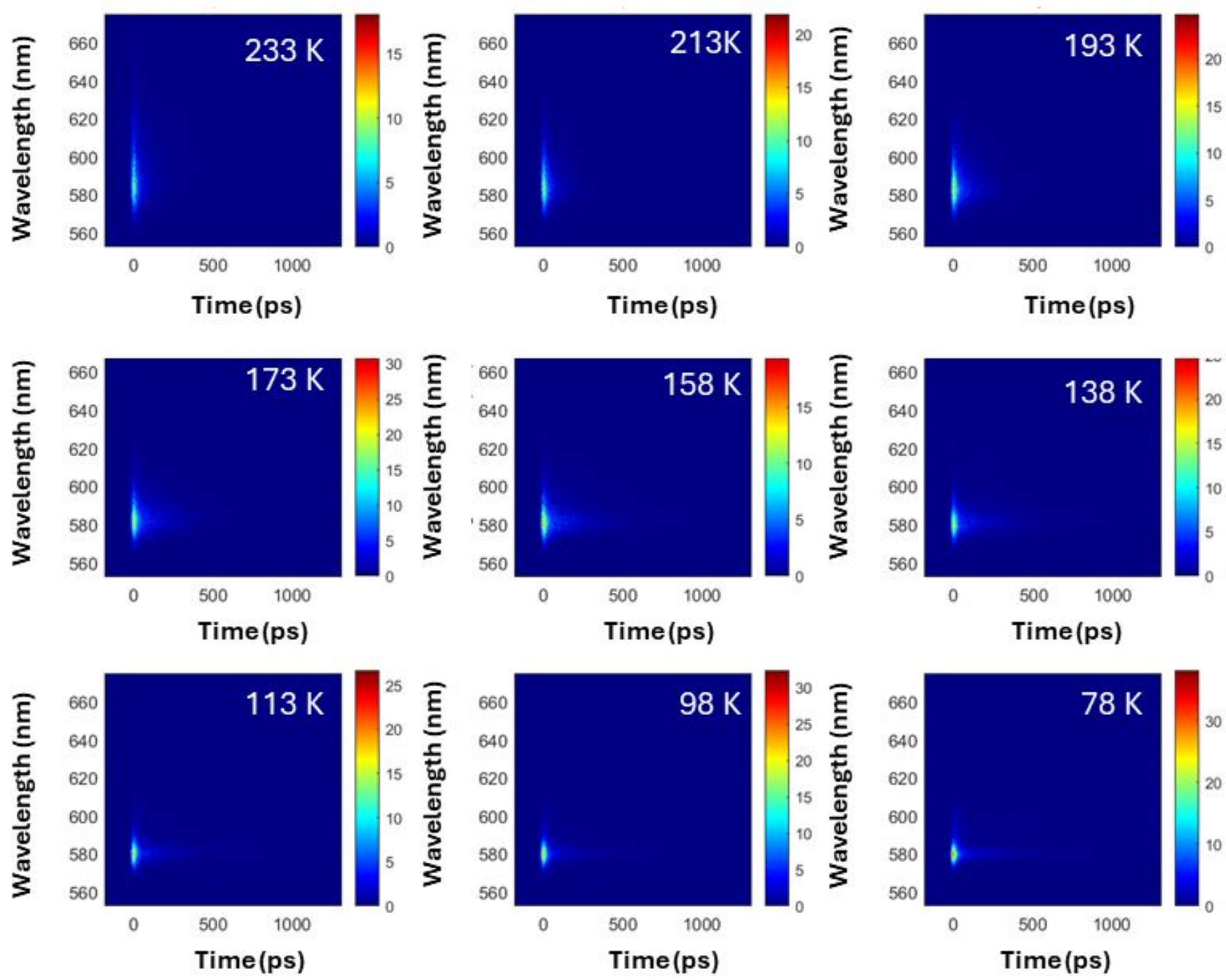

Figure S19: Time Resolved Photoluminescence at different temperatures of BA_2SnI_4, performed with an excitation wavelength of 515 nm, 80 MHz, Power out 0.68 µW (0.140 on samples), with a grating of 150 gr/mm

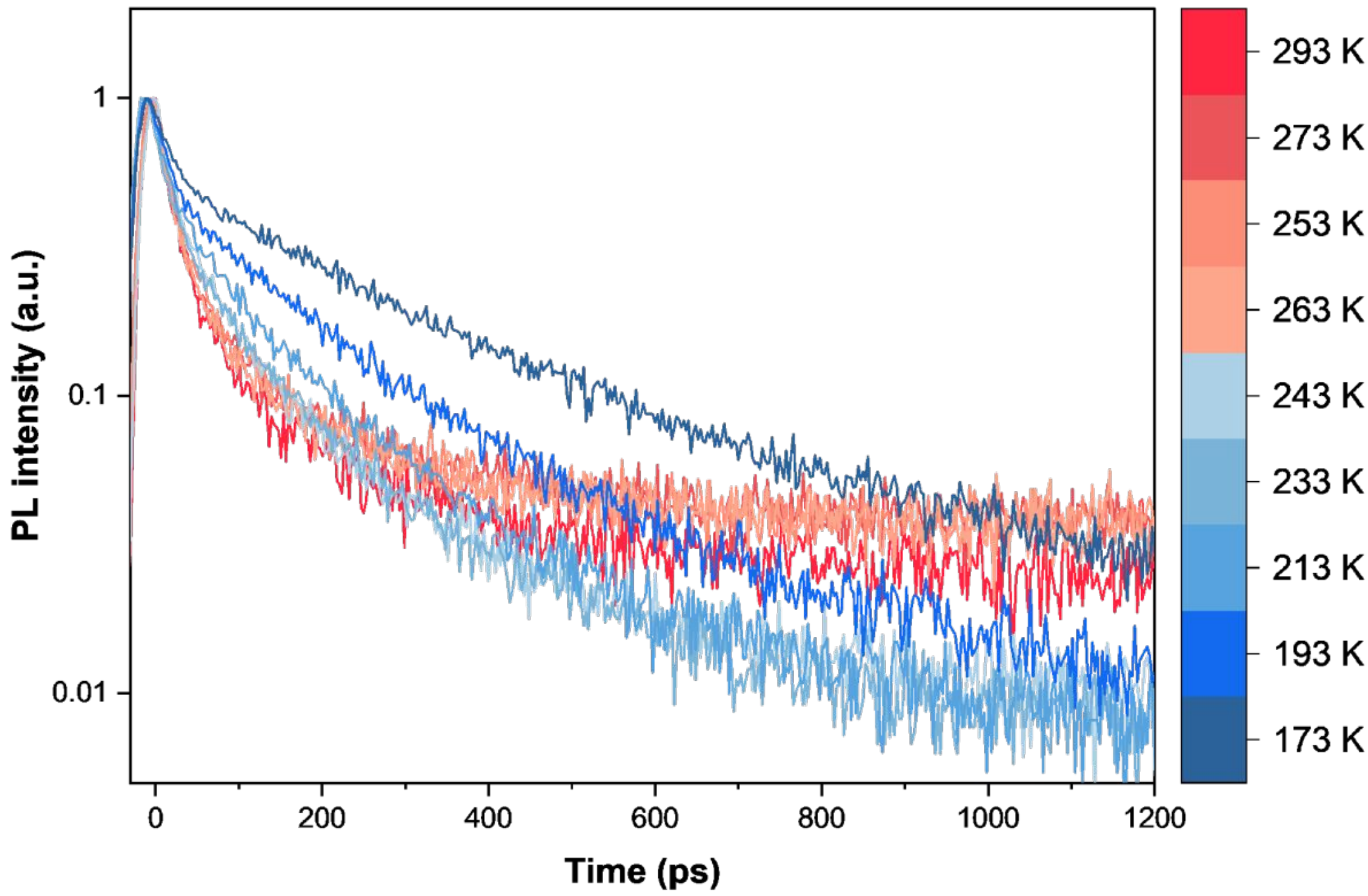

Figure S20: TRPL decay of BA_2SnI_4 in the temperature range 173-293 K. At these temperatures, it is not possible to disentangle the contribution from excitonic and trap emission. Excitons possess sufficient thermal energy to overcome the energy barrier towards trapping. This results in continuous exchange between excitons and traps, leading the system toward a dynamic equilibrium where the observed photoluminescence is a close convolution of the two contributions. The blue curve corresponds to the low-temperature phase, while the red curve represents the high-temperature phase after the phase transition. A clear change in the lifetime can be observed after the transition.

4. Amplified spontaneous emission measurements

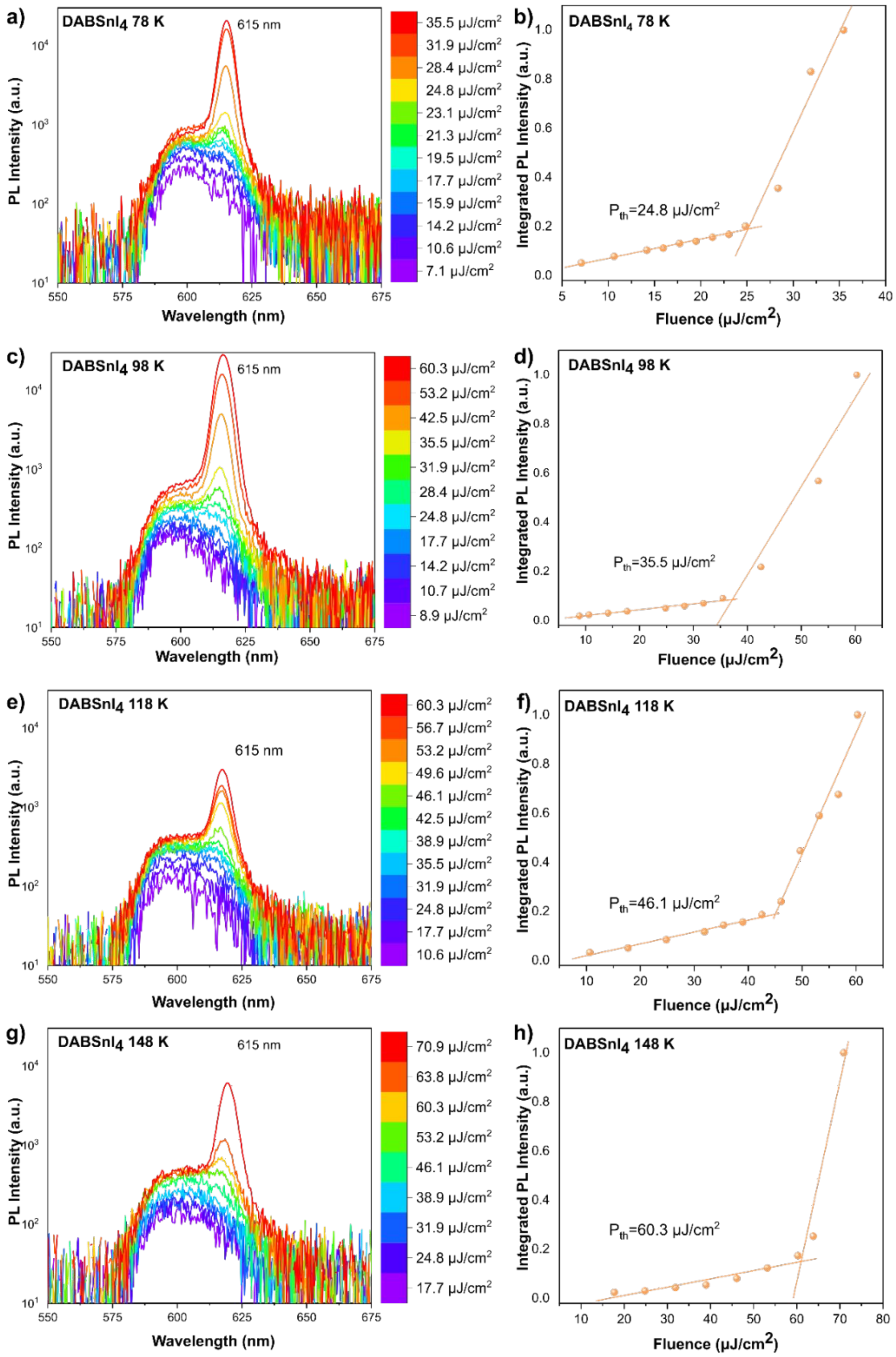

Figure S21: Amplified spontaneous emission (ASE) of $DABSnI_4$ at different temperatures along with the corresponding excitation fluence: a) ASE at 78 K, b) fluence at 78 K with a P_{th}=24.8 μJ/cm^2 , c) ASE at 98 K and d) fluence at 98 K with a P_{th}=35.5 μJ/cm^2, e) ASE at 118 K and f) fluence at 118 K with a P_{th}=46.1 μJ/cm^2, g) ASE at 148 K and h) fluence at 148 K with a P_{th}=60.3 μJ/cm^2.

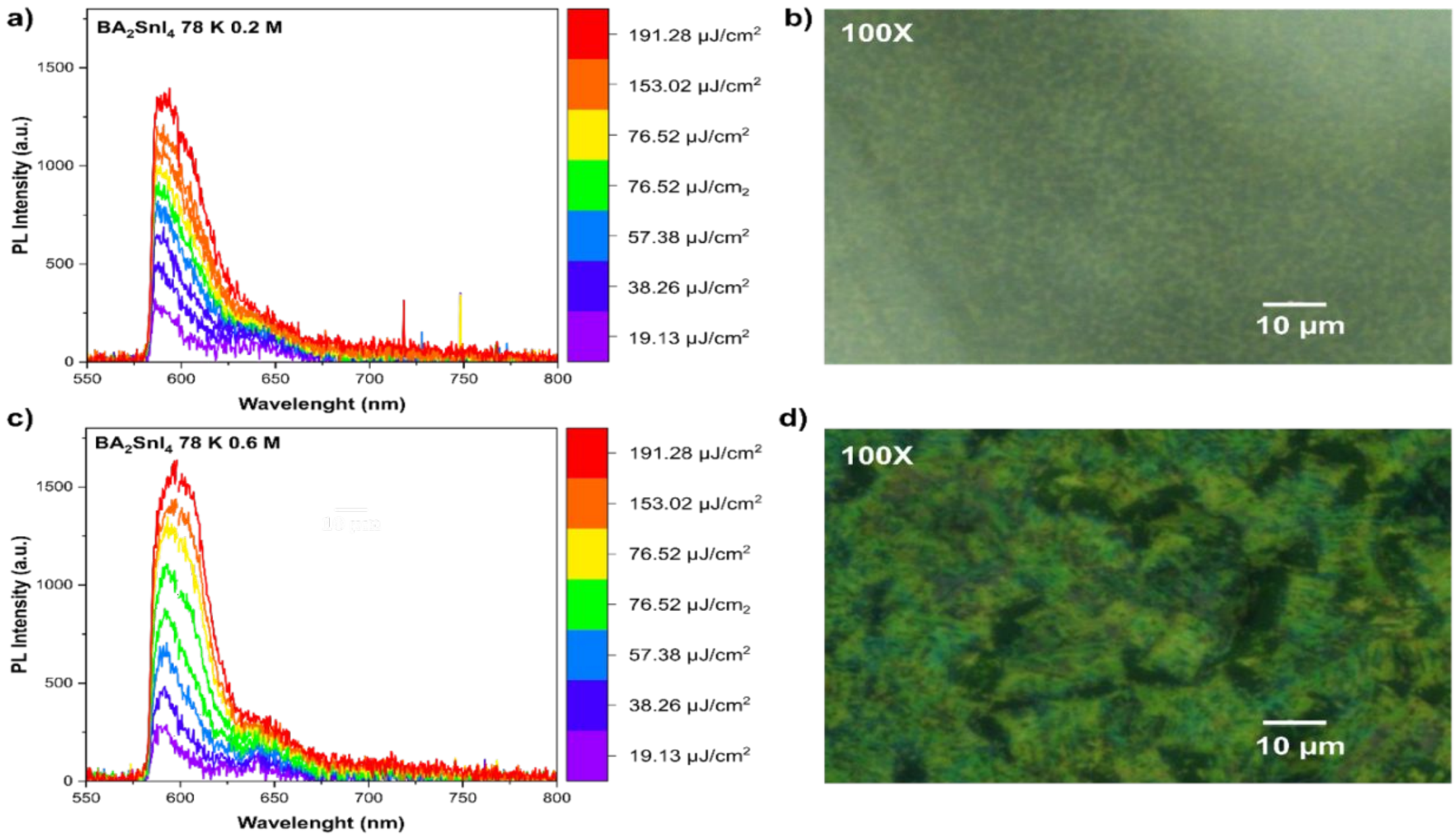

Figure S22: Fluence-dependent PL at 78 K and optical images of BA_2SnI_4: a) the ASE and b) optical image (x100 Magnification 100 x) of a 0.2 M thin film, while c) is the ASE and d) the optical image of a 0.6 M thin film. In both cases, the material doesn't show ASE, and the grains are less defined and smaller compared to $DABSnI_4$.

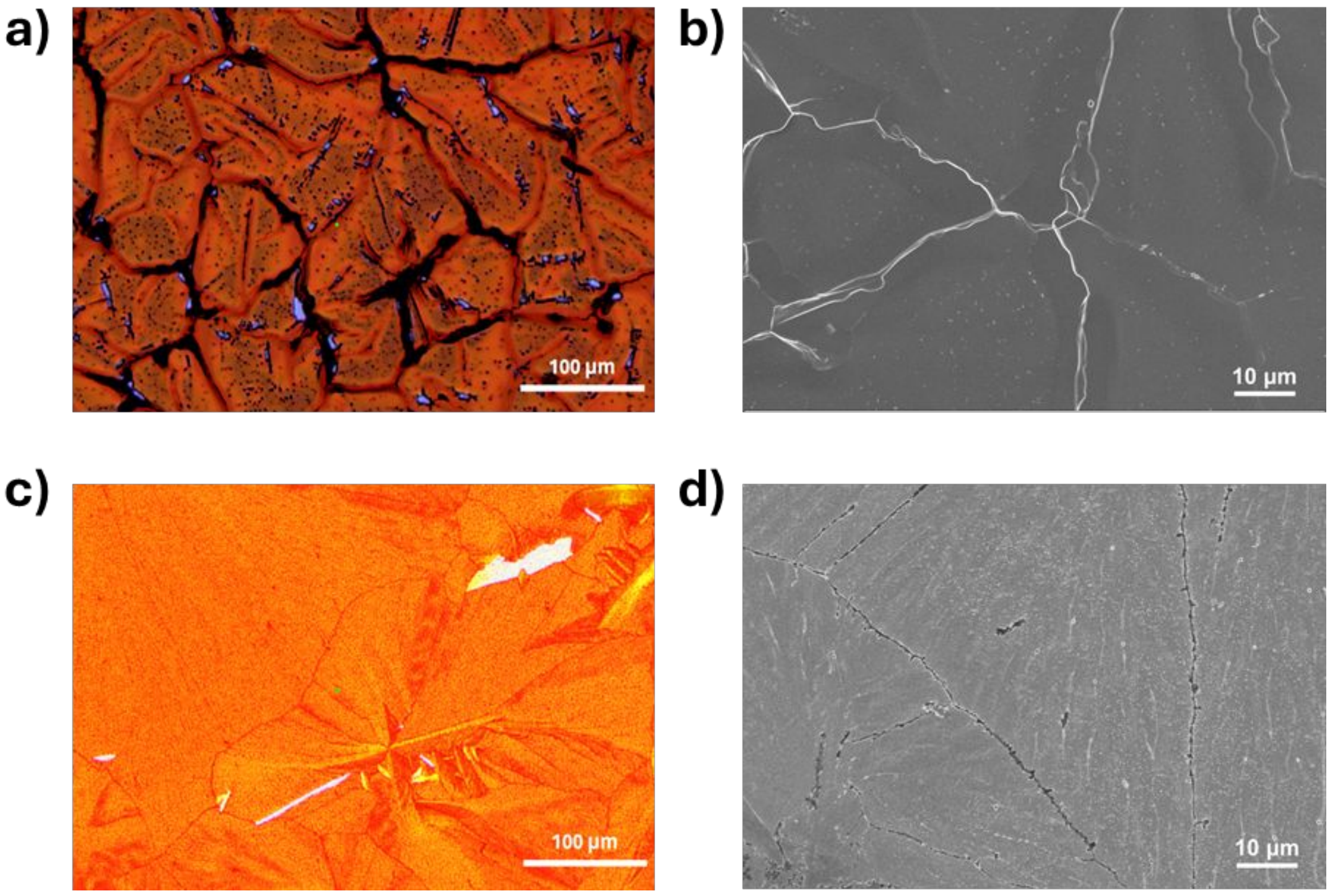

Figure S23: Optical microscope images (x20) of a) BA_2SnI_4 and c) of $DABSnI_4$. SEM images of b) BA_2SnI_4 (working distance 3.9 mm, Electron High Tension (EHT) of 3 kV, magnification 1.54k x) d) $DABSnI_4$ (working distance 4.5 mm, EHT 2 kV and magnification 1.10k x).

References

1. Smith, M. D.; Jaffe, A.; Dohner, E. R.; Lindenberg, A. M.; Karunadasa, H. I. Structural Origins of Broadband Emission from Layered Pb–Br Hybrid Perovskites. Chem. Sci. 2017, 8 (6), 4497–4504. https://doi.org/10.1039/C7SC01590A
2. Shao, Y.; Gao, W.; Yan, H.; Li, R.; Abdelwahab, I.; Chi, X.; Rogée, L.; Zhuang, L.; Fu, W.; Lau, S. P.; Yu, S. F.; Cai, Y.; Loh, K. P.; Leng, K. Unlocking Surface Octahedral Tilt in Two-Dimensional Ruddlesden-Popper Perovskites. Nat. Commun. 2022, 13 (1), 138. https://doi.org/10.1038/s41467-021-27747-x
4. Hartman, J. D.; Kudla, R. A.; Day, G. M.; Mueller, L. J.; Beran, G. J. O. Benchmark Fragment-Based 1H, 13C, 15N and 17O Chemical Shift Predictions in Molecular Crystals. Phys. Chem. Chem. Phys. 2016, 18 (31), 21686–21709. https://doi.org/10.1039/C6CP01831A
5. Brivio, F.; Sarsembek, N.; Martelli, G.; Scotillo, R.; Loprete, F.; Angelis, F. D.; Cortecchia, D. Discerning Two-Dimensional Metal Halide Perovskite Moieties Using Solid-State NMR Fingerprints. J. Mater. Chem. A 2025, 13 (39), 33739–33748. https://doi.org/10.1039/D5TA02747K
6. Bloembergen, N.; Purcell, E. M.; Pound, R. V. Relaxation Effects in Nuclear Magnetic Resonance Absorption. Phys. Rev. 1948, 73 (7), 679–712. https://doi.org/https://doi.org/10.1103/PhysRev.73.679
7. Fabini, D. H.; Siaw, T. A.; Stoumpos, C. C.; Laurita, G.; Olds, D.; Page, K.; Hu, J. G.; Kanatzidis, M. G.; Han, S.; Seshadri, R. Universal Dynamics of Molecular Reorientation in Hybrid Lead Iodide Perovskites. J. Am. Chem. Soc. 2017, 139 (46), 16875–16884. https://doi.org/10.1021/jacs.7b09536
8. He, Y.; Wong, E. L.; Sarsembek, N.; Samanta, R.; Regaldo, D.; Pianetti, A.; Cecconi, M.; Lanzani, G.; Petrozza, A.; Cortecchia, D. Role of the Metal Cation on the Amplified Spontaneous Emission Properties of Two-Dimensional Perovskites. J. Am. Chem. Soc. 2025, 147 (48), 44175–44184. https://doi.org/10.1021/jacs.5c13296
9. Folpini, G.; Cortecchia, D.; Petrozza, A.; Kandada, A. R. S. The Role of a Dark Exciton Reservoir in the Luminescence Efficiency of Two-Dimensional Tin Iodide Perovskites. J. Mater. Chem. C 2020, 8 (31), 10889–10896. https://doi.org/10.1039/D0TC01218A
10. Srimath Kandada, A. R.; Silva, C. Exciton Polarons in Two-Dimensional Hybrid Metal-Halide Perovskites. J. Phys. Chem. Lett. 2020, 11 (9), 3173–3184. https://doi.org/10.1021/acs.jpclett.9b02342

11. Neutzner, S.; Thouin, F.; Cortecchia, D.; Petrozza, A.; Silva, C.; Srimath Kandada, A. R. Exciton-Polaron Spectral Structures in Two-Dimensional Hybrid Lead-Halide Perovskites. Phys. Rev. Mater. 2018, 2 (6), 064605. https://doi.org/10.1103/PhysRevMaterials.2.064605
